# Functional Amyloids and their Possible Influence on Alzheimer Disease

**DOI:** 10.15190/d.2017.9

**Published:** 2017-10-16

**Authors:** Angus Lau, Matthew Bourkas, Yang Qing Qin Lu, Lauren Anne Ostrowski, Danielle Weber-Adrian, Carlyn Figueiredo, Hamza Arshad, Seyedeh Zahra Shams Shoaei, Christopher Daniel Morrone, Stuart Matan-Lithwick, Karan Joshua Abraham, Hansen Wang, Gerold Schmitt-Ulms

**Affiliations:** ^1^Department of Laboratory Medicine & Pathobiology, University of Toronto, Medical Sciences Building, 6th Floor, 1 King's College Circle, Toronto, Ontario M5S 1A8, Canada; ^2^Tanz Centre for Research in Neurodegenerative Diseases, University of Toronto, Krembil Discovery Centre, 6th Floor, 60 Leonard Avenue, Toronto, Ontario M5T 2S8, Canada

**Keywords:** Alzheimer disease, amyloid, Aβ, somatostatin, oligomeric, interaction

## Abstract

Amyloids play critical roles in human diseases but have increasingly been recognized to also exist naturally. Shared physicochemical characteristics of amyloids and of their smaller oligomeric building blocks offer the prospect of molecular interactions and crosstalk amongst these assemblies, including the propensity to mutually influence aggregation. A case in point might be the recent discovery of an interaction between the amyloid β peptide (Aβ) and somatostatin (SST). Whereas Aβ is best known for its role in Alzheimer disease (AD) as the main constituent of amyloid plaques, SST is intermittently stored in amyloid-form in dense core granules before its regulated release into the synaptic cleft. This review was written to introduce to readers a large body of literature that surrounds these two peptides. After introducing general concepts and recent progress related to our understanding of amyloids and their aggregation, the review focuses separately on the biogenesis and interactions of Aβ and SST, before attempting to assess the likelihood of encounters of the two peptides in the brain, and summarizing key observations linking SST to the pathobiology of AD. While the review focuses on Aβ and SST, it is to be anticipated that crosstalk amongst functional and disease-associated amyloids will emerge as a general theme with much broader significance in the etiology of dementias and other amyloidosis.

## SUMMARY

1. *Introduction*
2. *What are amyloids and oligomeric aggregates?*
3. *Post-translational generation of Aß and its role in the etiology of AD*
4. *Oligomeric versus amyloid A* *ß* *in AD*
5. *Interactors of oligomeric and/or fibrillar A* *ß*
6. ** *Aβ strains, cross-seeding and co-aggregation*
7. *Functional amyloids*
8. *Biogenesis and physiological function of SST & CST*
9. *Evidence for direct interaction of SST*** *&*** *CST with Aß*
10. ** *Interactors of SST (and/or CST)*
11. *Levels and distribution of A* *ß* *versus SST (or CST) in the healthy brain*
12. *Changes to the expression or down-stream signaling of SST/CST in AD*
13. *Evidence for co-localization of SST and CST with plaques and NFTs*
14. *Conclusion*

## 1. Introduction

The misfolding and ensuing self-aggregation of proteins is intimately linked to the development of over forty human diseases with diverse etiologies and clinical characteristics^1^. These include a panoply of neurodegenerative disorders, ranging from relatively rare albeit fatal prion disorders, such as Creutzfeldt-Jacob disease, to prevalent conditions like Alzheimer disease (AD). Typically, pathological protein aggregation in these disorders is characterized by the transformation of an ordinarily soluble and functional protein into insoluble, highly-ordered, fibrillar deposits, frequently referred to as amyloids. Because of the widespread nature and implications of this phenomenon for human health, efforts to better understand protein misfolding and aggregation have grown dramatically in the past twenty years. Indeed, this body of work has yielded a number of exciting developments, including the recognition that the self-assembly of disease-associated proteins can spawn a heterogeneous population of smaller oligomeric intermediates. These smaller assemblies fulfill crucial roles as both precursors of mature amyloids, and as cytotoxic effector,s independently capable of triggering cellular dysfunction^2,3^.

One of the best understood amyloidogenic peptides is amyloid β (Aβ), known to accumulate in the brains of individuals afflicted with AD^4^. Not only are the steps underlying its biogenesis thoroughly investigated but there also is extensive knowledge related to precise conditions that can promote the formation of oligomeric, pre-fibrillar and fibrillar assemblies of this peptide^5^. There also is no shortage of reports describing molecular interactions of Aβ^6^. However, this aspect of its biology remains less well understood due to a shortfall in studies that take a broad and unbiased approach to discovery in their investigation of Aβ interactions^7^. It is increasingly apparent that the propensity of Aβ to give rise to toxic assemblies is influenced by its interactions and depends on cellular receptors^8^. It also is apparent that the precise conditions that promote the formation of small aggregates on the way to forming toxic assemblies can give rise to more than one conformer^9^. Whereas such ‘strain’ phenomena have been under intense scrutiny in the prion field for many years, they are still somewhat new to the broader field invested in the study of amyloid disorders, despite their potential significance for the pathobiology of several of the most prevalent neurodegenerative diseases. Further adding to the complexity of the amyloid landscape are an ever growing number of proteins understood to acquire amyloid characteristics under normal conditions^10^. These natural amyloids are not confined to only exotic paradigms; natural amyloids are also observed in the brain, where they may, for example, play roles in memory consolidation or the temporary storage of peptides in dense granules prior to their release into the synaptic cleft. The latter type of amyloid is prominently represented by a subset of neurohormones, including the cyclic neuropeptide SST^11^. The co-existence of natural amyloids has largely been ignored in the context of amyloid disorders. However, their presence generates a potential reality, whereby there can be spatial overlap and crosstalk in the form of cross-seeding or coaggregation of different types of amyloidogenic proteins.

In support of this scenario, we recently identified SST in an unbiased search for Aβ interaction partners as the peptide that most selectively interacted with oligomeric Aβ (oAβ) but not monomeric Aβ (mAβ)^12^. We then observed that SST can influence the aggregation characteristics of Aβ in an assay undertaken at physiological pH and salt conditions. Finally, we were able to visualize direct binding and the formation of distinct oAβ complexes, which only formed in the presence of SST. Based on these results, we were intrigued to take stock of what is known about the relationship of SST and Aβ. Whereas the direct interaction between these peptides had, to the best of our knowledge, not been reported before, overlaps in the biology of SST and Aβ had been noted. In fact, several manuscripts pointed toward some role of SST in the etiology of AD by documenting that (i) SST-releasing neurons are often observed in spatial proximity to plaques^13^, (ii) levels of SST receptors are reduced in AD^14^, (iii) SST expression levels decline with age, and even more pronouncedly in AD, (iv) binding of SST to its receptors triggers a signaling cascade, which controls the release of proteolytic enzymes involved in the degradation of Aβ^15^, and (v) genetic variants within the SST gene locus alter the risk for AD^16,17^.

This article was written with the intention to review this body of literature and shine a light on the possibility that SST may also influence the etiology of AD on account of its direct interaction with Aβ. To this end, the first set of chapters will introduce the role of oligomeric assemblies in the pathobiology of neurodegenerative diseases and recap the biogenesis of Aβ and its oligomeric assemblies. This will be followed by a brief summary of Aβ interactors. We will then remind the reader of concepts related to crosstalk amongst amyloidogenic proteins, and introduce functional amyloids, focusing on SST, its biogenesis and known interactions. Subsequent chapters will review in more detail the evidence for SST binding to Aβ, compare the distribution of these peptides in the brain, and critically explore prior knowledge which connected SST to Aβ in the context of AD.

## 2. What are amyloids and oligomeric aggregates?

### 2.1 Historical perspective

The term “amyloid” was first employed in medicine in the 1850s to describe large pathological tissue deposits found in a variety of seemingly unrelated disorders^18^. The use of the term “amyloid”, meaning ‘starch-like’, was based initially on an erroneous belief that deposits were composed of carbohydrate, owing to their starch-like affinity for iodine in the presence of sulphuric acid^19^. Despite its etymological origins, the term “amyloid” was retained when it was eventually discovered that deposits were in fact proteinaceous in nature^19^. Over a century of concerted efforts to define the structure of proteinaceous amyloid deposits in disease followed, with progress largely reflecting broader advances in biochemistry and physics. This included the discovery of amyloid binding to histochemical dyes like Congo red^19^, the ultrastructural visualization of amyloid fibrils using electron microscopy (EM)^20^, and the isolation and analysis of protein aggregates from cases of “primary amyloidosis”, which culminated in the identification of human immunoglobulin light chain as the first amyloidogenic protein^21^. Although amyloids were mostly studied in the context of disease, the application of modern molecular biology and biophysics in the 1990s led to the recognition that a much larger number of non-disease-associated proteins could also undergo self-aggregation under suitable experimental conditions^22^. In fact, it is now well-known that proteins can adopt a number of conformational states ranging from common monomeric soluble species to various functional and non-functional aggregated forms. The amyloid state refers to aggregates of highly-ordered, fibrillar, and beta-sheet-rich assemblies of polypeptide chains. Unlike the native fold of a protein, whose architecture is strongly influenced by the unique properties of side-chains encoded in the amino acid sequence, the amyloid state relies predominantly on generic peptide backbone characteristics and, therefore, is accessible to almost any polypeptide chain. Thus, the contemporary definition of amyloids is situated within a thermodynamic landscape of protein states, and comprises both proteins in their pathological and normal states.

### 2.2 Physicochemical characteristics of amyloids

It follows that amyloid aggregates originating from different constituent proteins exhibit many shared biochemical, biophysical and ultrastructural attributes^23^. Most often, when visualized by electron microscopy (EM) or atomic-force microscopy (AFM) they are observed as non-branching rope-like fibrils that are 7.5-10 nm in diameter and 3-100 mm in length^19^. Individual fibrils are themselves comprised of 2-6 protofilaments twisted around each other, with the core of each protofilament consisting of stacked beta-strands oriented perpendicularly to the fibril axis^23,24^. This structural arrangement produces the characteristic cross-b pattern seen in X-ray fibre diffraction analyses and confers a high degree of kinetic and thermodynamic stability to amyloids. In reality, the propensity of proteins to form amyloids under physiological conditions differs widely. Intriguingly, evolution has more than once independently harnessed this propensity, thereby achieving favourable outcomes directly related to these properties^11^.

### 2.3 Amyloids in neurodegenerative diseases

Amyloid deposition of a particular protein is pathognomonic for a number of neurodegenerative diseases^25^. For example, senile plaques in AD and Lewy inclusion bodies in Parkinson disease (PD), are composed of pathologically aggregated amyloid beta peptide (Aß) and alpha-synuclein (α-SN), respectively. The conversion of ordinarily active, soluble proteins into misfolded states with abnormal propensity for self-aggregation is a multifaceted disease-specific phenomenon^1^. Triggers for conversion may arise from diverse sources and include age-related changes to the levels of amyloidogenic proteins (Aβ in AD), predisposition of proteins to adopt pathologic conformations (e.g., α-SN in sporadic PD), genetic alterations that augment the amyloidogenic propensity of proteins (e.g., polyglutamine expansion of the *HTT *gene in Huntington’s disease) and abnormal post-translational modifications (e.g., Tau hyperphosphorylation in AD)^1^.

The predominant view in the past was that mature amyloid fibrils (i.e., macroscopic aggregates) represented the main perpetrators of neuronal death^25^. However, this model of pathogenesis is weakened by the detection of amyloid deposits in the brains of cognitively normal individuals^26,27^, and by a lack of correlation between disease stage/ progression and amyloid burden^28^. In fact, evidence has been mounting that prefibrillar oligomeric species, and not macroscopic fibrillar deposits, represent the predominant toxic entities in several neurodegenerative disorders, including AD, PD, and prion diseases^25^.

It is now well-established that amyloid formation represents a complex, dynamic, context-dependent, multi-step process capable of yielding a heterogeneous population of smaller oligomers, in addition to macroscopic mature amyloid fibrils^25^. Numerous oligomeric species within the aggregation pathways of several disease- and non-disease-associated proteins including Aß, α-SN, the prion protein (PrP), islet amyloid peptide, HypF-N, and fascicilin 1-4 have been identified^25,29,30^. Pre-fibrillar oligomers can serve as direct precursors to mature amyloids (on-pathway), or exist as so-called off-pathway aggregates^1^. Importantly, there is compelling *in vitro *and *in vivo* evidence for pronounced cytotoxicity of oligomers, independent of their role as amyloid precursors^2,3,31^. The realization that levels of soluble oligomers correlate more directly with disease progression provides an impetus to better characterize the structural and biochemical basis for oligomer-mediated toxicity^28^.

In contrast to their amyloid counterparts, less is known about the structural properties of oligomeric assemblies. High-resolution, atomic-level characterization of oligomers is complicated by the inherently transient and heterogeneous nature of these aggregation intermediates^25^. Nevertheless, techniques such as EM, atomic force microscopy (AFM), hydrogen-deuterium exchange and fluorescence spectroscopy have been used to generate low-resolution structural models^29,32^. Oligomeric intermediates display tremendous heterogeneity in morphology and size. For instance, oligomeric α-SN can exist as dimers, spheres, chains of spheres, rings, and doughnut-shaped assemblies^25,30,32^. A similarly diverse spectrum of morphologies has been reported for oligomeric Aß that will be discussed in-depth in subsequent sections^24,25,29^. Importantly, oligomeric species tend to exhibit a higher propensity for surface-exposed hydrophobic patches compared to monomeric or amyloid fibril counterparts, a property that may also represent a key structural determinant of oligomeric cytotoxicity^33^.

### 2.4 Amyloid-based cellular toxicity

Uncovering precise mechanisms by which oligomeric species mediate their toxic effects remains a chief priority in protein misfolding and neurodegenerative disease research. A number of potential hypotheses have been proposed, including oligomer-driven sequestration and incapacitation of crucial cellular proteins, activation of pro-apoptotic signaling cascades, and enhanced oxidative stress due to the generation of free-radical species^25^. An emerging mechanism with mounting evidence is the disruption of lipid membranes via aberrant, lipid bilayer-oligomer interactions. The use of specific α-SN oligomers displaying superior stability over a range of pH, temperatures, and incubation conditions, has provided a useful model to investigate this concept experimentally^30,34^. An example of one such α-SN oligomer is an ellipsoidal assembly composed of 30 monomers, which exhibits a structured, ß-sheet-rich core, surrounded by a disordered shell^30,34^. Vesicle leakage experiments have revealed that these α-SN oligomers are far more potent at inducing membrane permeabilization than monomeric forms, although both are capable of interacting with lipid membranes. Several studies indicate that oligomeric α-SN aggregates interact with the lipid bilayer through both exposed hydrophobic patches and N-terminal domain features^30,33^. However, the precise mechanisms by which such interactions lead to membrane disruption remain unclear. The future elucidation of higher resolution structures should help clarify these mechanisms. Insights into structure-toxicity relationships have also benefited greatly from studies employing non-human, aggregation-prone proteins, such as the bacterial HypF-N protein derived from *Escherichia coli*. Two HypF-N oligomeric intermediates can be generated under different experimental conditions^35,36^ Intriguingly, these oligomers are indistinguishable in terms of morphology, stability, and binding propensity to lipid membranes but possess strikingly dissimilar cytotoxic properties. The basis for this difference appears to be related to the degree of surface exposure of hydrophobic patches, which ultimately correlates with membrane penetration potential and overall toxicity^35,36^. Thus, a recurring theme of hydrophobic accessibility may represent a generalizable and potentially targetable mechanism of toxic, disease-associated prefibrillar oligomers. Continued efforts at achieving detailed structural and biophysical characterization of the aggregation pathway, as well as the identification of various endogenous modulators of this process, will be imperative in the rational design of effective therapeutics.

## 3. Post-translational generation of Aß and its role in the etiology of AD

### 3.1 Endoproteolytic genesis of Aß 

One of the earliest events in the pathogenesis of AD is the aggregation of Aß, a small hydrophobic peptide that is generated through the sequential proteolytic processing of the larger amyloid precursor protein (APP) by enzymes referred to as α-, ß-, and γ-secretasesw^37-40^. The amyloidogenic pathway, responsible for the generation of Aß, relies on the initial processing of APP by ß-secretase. The term ß-secretase designates two specific Type I transmembrane aspartyl proteases, BACE1 and BACE2^41^. Although both BACE enzymes function best at slightly acidic pH, they are differentially expressed in the body, with BACE1 being highly expressed in neurons and BACE2 being predominantly found in peripheral tissues. Processing of APP by BACE1 generates a large secreted ectodomain fragment, sAPPb, and a C-terminal fragment, composed of 99 amino acids (CTF99). Aß is released when CTF99 is further cleaved by γ-secretase, a heterotetrameric transmembrane complex that relies on one of two presenilin intramembrane aspartyl proteases for mediating the cleavage^37,42-45^.

Given that ß-secretase functions best at acidic pH, endosomes have been proposed to be a critical cellular compartment for BACE1 processing of APP^46^. Once there, APP can be targeted to lysosomes for degradation or shuttled back to the plasma membrane. The functional γ-secretase complex has been detected at several post-endoplasmic reticulum (ER) compartments, including the TGN, endosomes and the plasma membrane^47^. Therefore, once APP is processed by BACE1, it may be further endoproteolyzed to give rise to Aß in more than one subcellular compartment^48-52^.

### 3.2 Significance of Aß variants in AD

Close scrutiny has revealed that the amyloidogenic processing of APP can give rise to several Aß variants. More specifically, BACE1 can cleave APP primarily at two distinct residues, yielding membrane-bound C-terminal fragments CTF89 and CTF99^41^. The subsequent cleavage by γ-secretase is even less precise, yielding cleavage products differing in their C-terminal boundaries. These products conform to designations, such as Aß_1-X _and Aß_11-X_,with x representing Aß residues 37-43^47^. Despite this heterogeneity, Aß_1-40_represents the predominant cleavage product in the cerebrospinal fluid (CSF), followed by Aß_1-42_ and Aß_1-38_. The remaining Aß variants may make up no more than 1% of total Aß observed in the CSF^53^. The longer Aß_1-42_ peptide is more hydrophobic than shorter Aß peptides, and was observed to be the dominant Aß species found in senile plaques^54^. The two additional C-terminal amino acid residues within Aß_1-42_ were observed to stabilize specific neurotoxic oligomers not formed by Aß_1-40^55^_.

Further adding to the complexity of C-terminal Aß cleavage events may be the observation that γ-secretase complexes are composed of distinct presenilin or Aph-1 isoforms and that the subcellular localization of these isoforms may directly influence the relative production of Aß variants^56^.

Aß can undergo additional N-terminal truncations through the actions of proteases, including aminopeptidase A^57^. These N-terminally truncated Aß species make up a significant amount of the overall Aß pool associated with senile plaques^57^, perhaps reflecting the fact that N-terminal truncations render the peptide more hydrophobic overall and, thus, more prone to aggregation. Moreover, specific Aß residues can be subject to post-translational modifications, including oxidation, nitration, phosphorylation, pyroglutamylation and isomerization^57^. These modifications can, in turn, modulate the toxicity, aggregation and/or clearance of Aß and, therefore, may directly influence the pathogenesis of AD. For example, phosphorylation at serine residue 8 was shown to affect its toxicity^58^ and pyroglutamylation at positions 3 (3pE-Aß) or 11 (11pE-Aß) was observed to increase the propensity of Aß to aggregate^59^.

### 3.3 Aß cascade hypothesis

The accumulation and aggregation of toxic Aß in the brain has long been thought to be the trigger for downstream pathological processes, including the hyperphosphorylation of tau, that eventually lead to neuronal death in AD^60^. This “amyloid cascade hypothesis” is supported by genetic evidence that has established causative relationships of mutations in the APP gene and presenilin genes in familial cases of early-onset dementia^61-63^. Because of striking similarities in the pathological manifestation of early- and late-onset AD (LOAD), the hypothesis may have wider significance, i.e., beyond familial forms of the disease. Over the years, additional evidence in support of the amyloid cascade hypothesis has emerged^64^, including protective mutations found in the *APP* gene that can confer resistance against AD^65^ and gene products of several LOAD risk genes identified by genome-wide association studies (GWAS) that influence Aß processing, trafficking or clearance^5^. It also has become apparent that early changes to the brain’s Aß homeostasis, which lead to its accumulation and aggregation, may precede the onset of clinical symptoms by many years^66^. Once formed, Aß_42 _fibrils could accelerate the formation of neurofibrillary tangles (NFT) in transgenic mice expressing mutant tau protein^67^. However, the relationship between Aß and tau has been the subject of extensive debate and remains contentious, in part because NFTs and Aß deposition initiate at spatially distinct areas of the brain and Aß deposition has been found in cognitively normal elderly individuals^68^.

## 4. Oligomeric versus amyloid A in AD

### 4.1 Relative toxicity of Aβ conformers

Understanding the main drivers of AD toxicity has proven a difficult goal, and until the turn of the century, the prevalent view was that amyloid fibril neuropathology represented a precursor to cellular toxicity. Increasing evidence that soluble Aß, and not insoluble fibrils, correlated with neuronal loss shifted focus from Aß fibrils to Aß oligomers^28^. Since then, it has become apparent that although Aß fibrils can leak oligomers to some extent, the amyloid-Aß structure itself can be protective^69^. However, recognizing the importance of oligomers for the pathology of AD has added complexity. Although Aß oligomers seem to broadly impair memory functions when tested with both *in vivo *and *in vitro* models, molecular investigations into Aß toxicity have implicated a dizzying array of mediators. Two general paths to Aß toxicity, receptor ligation and membrane disruption, dominate the literature, but delineating the relative contributions and precise molecular underpinnings has proven difficult^70^. It is uncertain whether the toxic effects of an oligomeric Aß preparation manifest through promiscuous interactions, reflecting cumulative perturbations to the cell, or whether Aß toxicity acts predominantly through a single toxic mechanism. The deconvolution of this process has been hampered by the use of non-standardized Aß preparation protocols, and the observation that even precise protocols lead to heterogeneous mixtures of Aß assemblies.

For example, the first report describing the preparation of Aß-derived diffusible ligands (ADDLs) documented that these preparations inhibit the basic memory forming process, known as long-term potentiation (LTP), independent from N-methyl-D-aspartate receptor (NMDAR)-antagonists^71^. While still using the term ADDL, more recent reports were based on altered protocols for Aß preparation that made use of the solvents hexafluoroisopropanol (HFIP) and dimethyl sulfoxide (DMSO) as well as different Aß concentrations, limiting comparison across papers. It is important to note that non-physiological Aß concentrations, often in the high micro- to low milli-molar range, are routinely used and that this may lead to more promiscuous interactions, implicating less relevant mechanisms. Attempts to study Aß toxicity in the context of physiological pico-molar concentrations have suggested that oligomeric assemblies cause calcium dysregulation through a general membrane interaction and not through localized pore or receptor-mediated mechanisms^72^. The field is gradually adapting to standardized protocols and nomenclature, but more work in this direction is needed. Conformational antibodies have helped further this goal, and the mutually exclusive A11 and OC antibodies that detect pre-fibrillar oligomer (PFO) and fibrillar epitopes respectively have facilitated classifications^73^.

One currently unexplained observation is the strikingly reduced toxicity encountered with synthetic, as opposed to brain-derived, Aß_1-42^74-76^_. To uncover the molecular nature of the most profoundly toxic Aß preparations, the characterization of distinct assemblies remains a vital goal. Information such as Aß variants in use (Aß_1-40_ vs. Aß_1-42_), Aß source (synthetic or *in vivo)*, preparation protocol, and size of an assembly can help bridge the informational gap in lieu of high-resolution data. Intermediates along the path to fibrillogenesis (on-path assemblies) can be generally categorized as low and high mass pre-fibrillar oligomers. While no set limit delineates these two classifications, we will consider oligomers up to 100 kDa to be low mass as this includes the upper limit for PFOs and larger 18-mer Aß assemblies^77-79^. Generally, high mass assemblies seem to exert lower toxicity than their related protofibril or low mass counterparts^80,81^ and are less likely to exist at physiological concentrations, where higher order oligomers are rare^72,82^.

The aqueous phase of AD brain homogenates has been shown to impair memory associated functions in primary hippocampal slices. Immuno-captured preparations of Aß dimers, devoid of large oligomers following size-exclusion chromatography, were reported to retain the ability to inhibit LTP, enhance long-term depression (LTD), and reduce spine density when administered to a normal rodent hippocampus^80^. Through the use of antagonists, this spine loss was shown to depend on NMDAR function, and LTD enhancement to depend on metabotropic glutamate receptor 5, both receptors linked to synaptic plasticity. This same group designed AßS26C mutations, which tether pairs of Aß together through a disulfide bond, circumventing the need to work with less defined Aß dimers purified from brain samples. Even in the absence of brain-derived factors, these synthetic AßS26C dimers display toxicity^80^ and can induce Tau phosphorylation profiles resembling those observed in AD^76^. However, follow-up experiments partially disagreed with these results, as AßS26C dimers exhibited only mild toxicity following solubilization^83^. Incubation of AßS26C rapidly gave rise to larger assemblies and the authors proposed that these larger assemblies may have accounted for the previously observed toxicity.

### 4.2 Predominant oligomeric forms and their distinguishing features

Several groups have identified the primary toxic oligomers as Aß 6-mers that can stack into 12-mers. Initially described as synthetic Aß_1-42_-derived ADDLs, these 6-mers were found to assemble into 12-mer ADDLs at physiological temperatures and were detected in human AD-brains through the oligomer-specific, ADDL-raised M93 antibody^71,84^. More specifically, using photo-induced cross-linking of unmodified proteins (PICUP), Aß was observed to form 6-mer, 12-mer and 18-mer Aß assemblies, leading to the interpretation of the 6-mer as a ‘paranuclei’ or basic building block of other toxic Aß assemblies^78^. The use of PICUP to strengthen intermolecular interactions may have played a critical role in the identification of the 18-mer, as this Aß assembly is not as consistently identified by other groups. Interestingly, in contrast to Aß_1-42_, Aß_1-40_ did not assemble beyond the tetramer under identical experimental conditions^78^. Subsequent work by others established a protocol to preferentially form globular 12-mer assemblies, termed ‘globulomers’. Procedurally, this protocol resembled the aforementioned 2nd generation HFIP/DMSO protocol for generating ADDLs, but with the addition of 2% SDS or 0.5% fatty acid preparations to improve the stability of globulomers (final concentrations of 0.2% and 0.05% respectively)^85^. Taken together, these results corroborated the notion that 6-mer paranuclei represent the building blocks of 12-mer assemblies which, in turn, manifest as either on-path ADDLs or off-path globulomers, both capable of inhibiting LTP^71,85^.

Whereas the above assemblies are thought closely related and can be produced from synthetic Aß, a 12-mer assembly labeled Aß*56 has only been observed *in vivo *and appears to exhibit contrasting properties that underscore its uniqueness^86^. Although ADDLs, globulomers and Aß*56 have all been found in the brains of transgenic AD mice (Tg2576), their levels seem to differ throughout the course of ageing. Aß*56 was detected in Tg2576 brains at 6 months of age, with its levels remaining stable from there on^86^. Conversely, ADDLs and globulomers showed sharp increases in aged mice, beginning at 12 and 13 months respectively, with their levels continuing to rise until death^87^. In further contrast to ADDLs and globulomers, enriched preparations of Aß*56 only caused transient impairment of spatial learning when injected into Tg2576 mice. As Aß*56 has only been detected *in vivo, *a**necessity of brain-derived factors for its formation has been hypothesized^87^, but no such factors have been identified to date. Like ADDLs, Aß*56 has also been observed in human AD brains^84,88^, consistent with a potential relevance to AD.

### 4.3 Structural characterization of oligomeric and amyloid Aβ

**Figure 1:**
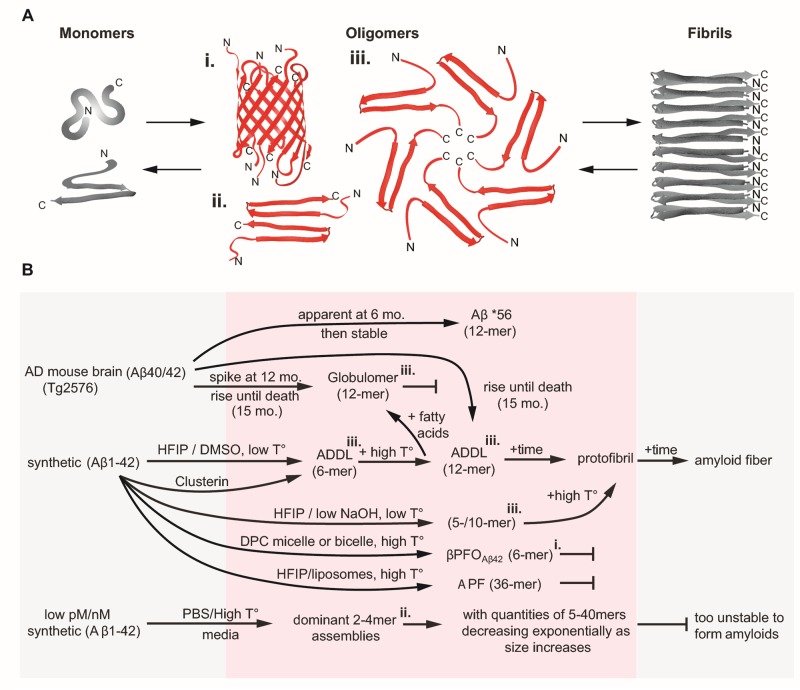
Fibrillogenesis and the conditions that promote specific Aß42 assemblies **A.** The general flow of fibrillogenesis begins with monomers, which assemble into oligomers, and eventual turn into amyloid protofibrils and fibrils. In addition to the canonical build-up indicated by arrows pointing right, oligomers, and even fibrils, may shed monomers (or oligomeric building blocks) depicted by reverse arrows. **B.** Flow-chart summaring major Aβ assembly pathways reported in the literature. Key features of the respective protocols are outlined in the flow chart. Roman numerals i.-iii. associate a given conformer with three-dimensional representations depicted in panel A^55,71,85,90,93^. 12-mer globulomers and ADDLs are associated with the identifier ‘iii’ as they’re hypothesized to form through stacking of planar 6-mers. 5-mer and 10-mer assemblies were also identified with ‘iii’ as they’re thought to form similar structures, albeit with one less protomer^55^. High temperature (T***°***) denotes a temperature of 37 ***°***C, low T***°*** denotes a range of 4-8 ***°***C. The abbreviation ‘mo.’ denotes months and DPC micelles/bicelles refer to dodecylphosphocholine micelles/bicelles. Red and black (or grey) colors designate toxic and innocuous molecular species, respectively.

Most mechanisms of Aß toxicity, whether specific or general, require interaction with the cell membrane and thus lipid interactions^70^. Annular Protofibrils (APFs) are an off-path, stable species that can form during the incubation of soluble PFOs with liposomes. APFs are 36-mer Aß assemblies that harbor spherical pores with internal diameters of 2.5-4 nm and that are hypothesized to cause Ca^2+^ dysregulation in cells^75^. Because APF-directed antibodies cross-react with a-hemolysin, they are thought to consist of a ß-barrel conformation. Pre-formed APFs are not toxic to cells and are reminiscent of bacterial b-barrel toxins that do not insert into the membrane pre-formed but must assemble in-membrane to exert their toxicity^89^. APFs are not the only ß-barrel assemblies of Aß, and a 6-mer Aß42-specific ß-barrel was found to assemble under optimal micelle or bicelle conditions. These ß-barrel pore-forming Aß42 oligomers (ßPFOs_Aß42_)­­ are estimated to carry an inner pore diameter of 0.7 nm and were shown to not only stably integrate into lipid bilayers but to induce 3 different patterns of pore conductance^90^. Although cytotoxicity has not been directly assayed for ßPFOs_Aß42_, their clear disruption of bilayer conductance and lower order assembly suggest that theyrepresent a more likely *in vivo* structure than APFs, and one with a more explicitly evinced mode of toxicity (**Figure 1**). Although *in vivo* reactivities of many of these assemblies have been validated, it bears repeating that lower pico- to nanomolar concentrations of Aß may preferentially produce lower order oligomers for both Aß1-42 and Aß1-40^72,82^. Under these more physiological conditions, the relative abundance of a given Aß oligomer seems to inversely correlate with its size, following an inverse exponential relationship where an Aß dimer would be 3000-fold more likely to form than a 10-mer^82^. The ability to study Aß assemblies at such low concentrations capitalizes on recent advances to single molecule microscopy techniques and relies on fluorophore-conjugated Aß preparations^91,92^.

Solid-state NMR, a tool that requires relative large analyte quantities, revealed structural constraints that may influence the oligomer-to-fibril transition. During the course of these studies, low salt and temperature conditions were observed to stabilize 5- and 10-mers, which can then proceed to fibrillize when gently agitated and incubated at 37 ºC^55^. This transition was found to occur in two steps. First, hydrophobic stretches comprising Leu17-Ala21 and Ile31-36Val undergo interstrand hydrogen bonding in a parallel, in-register fashion. Second, loosely packed oligomeric b-strands stagger to expose hydrophobic stretches for monomer addition^55^. This discovery of oligomer stabilizing conditions not only allowed the isolation of on-path oligomers, but suggested a simple mechanism to explain the shift from oligomer to fibril forming conditions, affording insight into the specific structural transitions necessary. The protomer conformation of Aß intermediate assemblies was further tested by creating a mutant Aß_1-42_CC that harbored two cysteine substitutions at residues 21 and 30, locking the oxidized monomer into a ß-hairpin^81^. By allowing Aß_1-42_CC to assemble in pro-fibril forming conditions, this work established that the ß-hairpin structure was conducive to oligomerization up to the protofibril stage. The lack of fibrillization and amyloid formation of Aß_1-42_CC was interpreted to suggest the necessity of a conformational shift away from ß-hairpin protomers during protofibril-to-fibril transition^81^.

Ion mobility shift mass spectrometry (IMS/MS) measurements^93^ revealed that Aß_1-40_ protomers shrink in size as oligomerization proceeds, while Aß_1-42_ protomers remain relatively unchanged^93^. The authors proposed that this distinction makes Aß_1-42_ more conducive to forming the globular 6-mers not observed with Aß_1-40^79^_. Taken together, it is apparent that complementary tools may be critical for deciphering the complexity of this assembly process. While the aforementioned conformational antibodies can help identify and catalogue various oligomeric assemblies, detailed structural characterization is needed to devise future, structure-specific inhibitors and imaging agents, and for elucidating the constraints that govern the interactions and toxicity of oligomeric Aß in AD.

## 5. Interactors of oligomeric and/or fibrillar Aβ

In light of its critical role in the pathobiology of AD, the identification of Aβ interactors has been an active area of research, and it is not surprising that several proteins were reported to bind to this peptide (reviewed in^6^). Here, we will describe studies that have reported factors which bind to oligomeric preparations of Aβ and confirm the binding of Aβ to small peptides.

### 5.1 Binders of oligomeric A*β *

A prevalent theme in this research has been the recognition that exposure of neurons to oligomeric Aβ preparations can perturb post-synaptic transmission by affecting the biology of receptors whose activity converges on and indirectly affects the NMDAR. For example, interactions of oligomeric Aβ with a7-nAChR have been proposed to mediate Ca^2+^ influx through the NMDAR^6^. The interaction has been proposed to lead to the recruitment of protein phosphatase PP2B and tyrosine phosphatase STEP, which together promote endocytosis of NMDAR^94^. Similarly, binding of Aβ oligomers to EphB2 is thought to promote the internalization and proteasomal degradation of this receptor tyrosine kinase, which has been proposed to trigger the co-internalization of NMDARs, thereby contributing to a decrease in LTP^95^. In recent years, Aβ oligomers were observed to interact with nanomolar affinity with the cellular prion protein^8^, leading to downstream activation of Fyn kinase with the involvement of mGluR5 and LRP1. It is thought that the phosphorylation of the GluN2B subunit of NMDAR by Fyn kinase leads initially to an increase in surface receptors, followed by their depletion and synaptic impairment^96^.

Other signaling platforms have been uncovered which involve direct binding of Aβ oligomers to receptors. One such example is the interception of Wnt3a binding to Frizzled by Aβ oligomers^97^. Canonical wnt signaling exhibits neuroprotective effects against Aβ oligomer toxicity. Neuronal insulin receptor function also can be disrupted by binding to soluble Aβ oligomers (ADDLs), an observation invoked to explain the association between Alzheimer disease and central nervous system (CNS) insulin resistance^98^.

### 5.2 Interactions of Aβ with small peptides

To our knowledge, the only naturally occurring peptides reported to bind Aβ are humanin and islet amyloid polypeptide (IAPP) (see Section 5). Humanin is a 24-amino acid peptide, encoded by the mitochondrial genome and found in the occipital lobe of AD patients^99^. *In vitro* studies with smooth muscle cells revealed that humanin exerts a protective effect on Aβ exposed cells, without affecting the relative abundance of Aβ or its propensity to form fibrils^100^. *In vivo *studies examining the consequences of injecting rats with humanin documented a humanin-dependent enhancement of LTP, possibly on the basis of it increasing dendritic branching and spine numbers^99^. Humanin-like compounds have since been developed, which have been shown *in vitro* and *in vivo* to have a stronger effect than the natural peptide itself in neuronal protection against Aβ toxicity. One example is [Gly14]-Humanin, which protects spatial learning and memory in rats against Aβ insult^101^.

Considerable research efforts have been invested in the characterization of synthetic Aβ-derived binding peptides. In particular, β-sheet breaker peptides represent a class of molecules that has garnered interest and features in several Aβ pre-clinical and clinical trials. These peptides were first introduced in 1996^102^, with one variant composed of Ac-LPFFD-amid (iAβ5) exhibiting protection against Aβ toxicity, preventing Aβ aggregation and even disassembling pre-formed fibrils^103^. These β-sheet breaking peptides are designed to resemble the Aβ core domain responsible for β-sheet formation but comprise prolines or methylated amino acids to impede the proper alignment of monomers during fibril extension^104^. Studies with mouse and rat models revealed that chronic intraperitoneal administration of iAβ5 can inhibit the formation of Aβ deposits, thereby improving the performance of rodents in memory and cognitive tasks^105^. The subsequent use of D-enantiomeric forms of this peptide, and the introduction of modifications that improved its ability to cross the blood-brain-barrier, further prevented its destruction by proteases, thereby enhancing its bioavailability^106^. This line of investigation has culminated in the development of compound PPI-1019 (Apan) (D-methyl-LVFFL), which has successfully completed Phase I and II clinical trials. Treatment of patients with mild-moderate AD with PPI-1019 led to increased levels of CSF Aβ, consistent with the interpretation that the compound enhanced clearance of Aβ from the brain^104^. Other examples of β-sheet breaker peptides include Trp-Aib (NH2-dTrp- α-aminoisobutyric acid -OH), which binds to low molecular weight Aβ assemblies, interferes with their toxicity and improves cognitive measures in rodent AD models^107^.

### 5.3 Interactions of Aβ with antibodies

A related strategy to designing peptide inhibitors, based on Aβ sequence motifs central to its aggregation, is to graft these sequences into the V (H) domain of antibodies^108^. A subset of these so-called gammabodies were observed to bind with nanomolar affinity to very specific conformations of Aβ, and did not exhibit cross-reactivity toward other polypeptides and amyloidogenic proteins. Specific examples of these gammabodies are those reliant on grafts of the VFFA or LMVGGVVIA motifs (comprising Aβ amino acid residues 18-21 or 34-42), which were shown to bind to Aβ fibrils or oligomeric Aβ, respectively, and are of interest mainly in AD research and diagnostic applications^108^.

Several other antibody-based Aβ binding reagents developed for the same purpose or for disease intervention trials were not derived by grafting but were raised traditionally by engaging the immune system of mice. One of them is specific to the Aβ_1–11_ residues on Aβ42. This antibody was shown to inhibit Aβ_1-42_ fibril formation and disaggregation of preformed fibrils^109^ but it did not bind to toxic Aβ oligomeric species, restricting its utility to preventative treatment approaches administered in a vaccine form with the objective to delay onset of disease^109^. A promising monoclonal antibody, known as Aducanumab, which was generated by Biogen Inc., has recently progressed into Phase 3 clinical trials. Aducanumab binds Aβ within the brain parenchyma and has been hypothesized to clear Aβ via a microglial phagocytic pathway. In an underpowered Phase Ib study patients who received a monthly intravenous dosage of Aducanumab showed a slowing of clinical decline^110^.

### 5.4 Interactions of Aβ with small molecules

Numerous small molecules have been screened and investigated for their effect on Aβ aggregation. Two important compound classes are polyphenols and membrane glycolipids. Polyphenols have been popular in Alzheimer’s research due to their existence in natural products. For example, epigallocatechin gallate (EGCG) is a compound found in green tea^111^ that has been shown to bind to Aβ in two different states, i.e., it interacts with the unfolded or monomeric Aβ and prevents its aggregation. Curiously, it also binds to mature Aβ fibrils, preventing their interaction with thioflavin T (ThT). Additionally, an inositol stereoisomer called scyllo-inositol stabilizes the non-toxic, low-n oligomers of Aβ, thereby preventing their accumulation into fibrillary conformers. scyllo-inositol has no effect, however, on pre-existing fibrils^112^.

## 6. Aβ strains, cross-seeding and co-aggregation

### 6.1 Aβ strains

**Figure 2:**
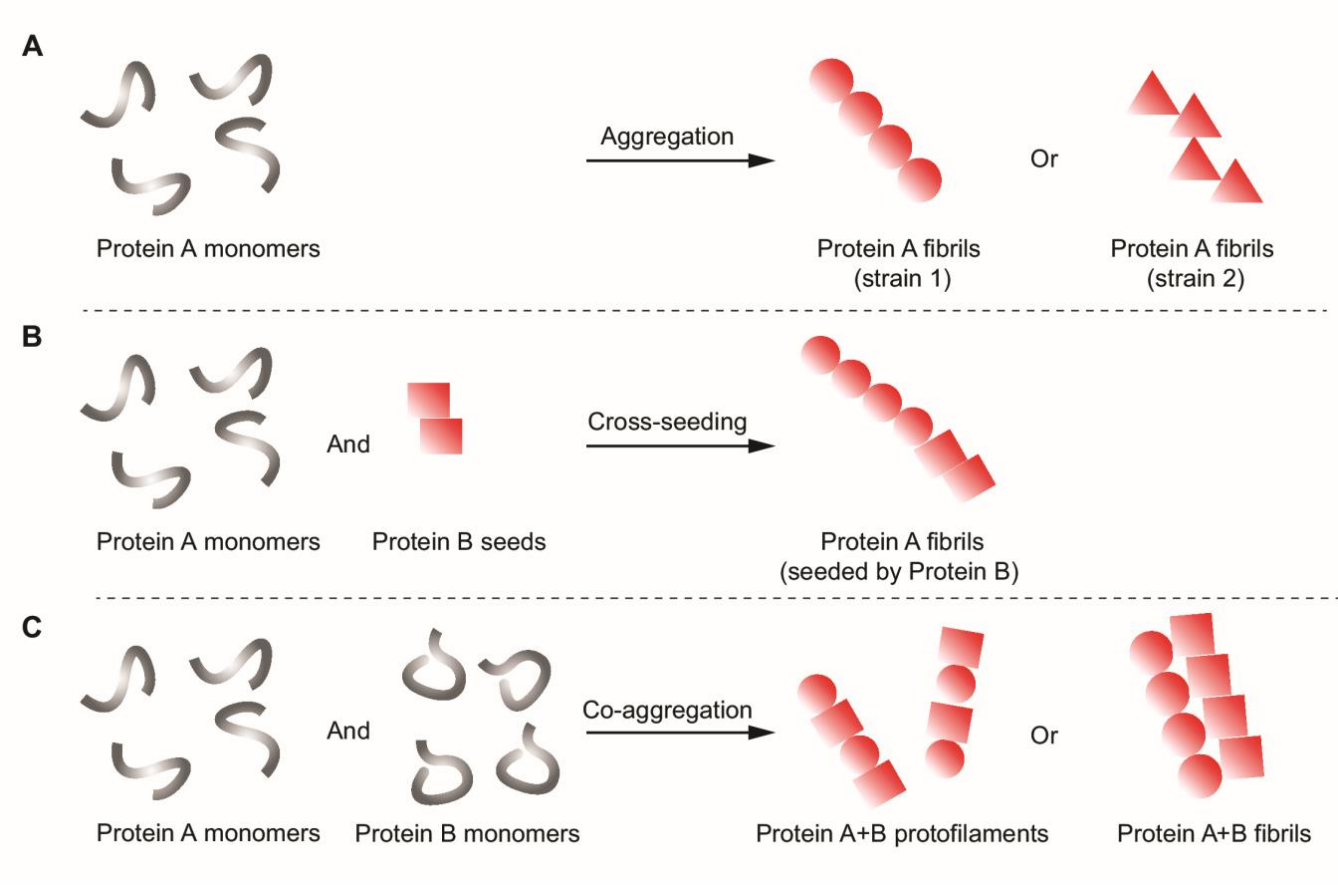
Strains, cross-seeding and co-aggregation. Cartoon depicting three related, yet distinct, concepts relevant to protein aggregation phenomena **A. **The same protein can give rise to distinct ‘strains’ of protein aggregates, if the arrangement of its monomers and the conformations adopted by the monomers within the aggregates are different. **B.** The term ‘cross-seeding’ designates a phenomenon whereby small aggregates composed of a given protein can seed the aggregation of another protein, thereby often influencing the kinetics and quaternary structure of aggregates forming. **C.** When two or more proteins ‘co-aggregate’ their monomers influence each other’s aggregation. Two separate scenarios can be further distinguished: the two different proteins can polymerize together and form mixed molecular species hybrid structures, or they can interact and influence each other’s aggregation, but polymerize separately to form single species structures.

It is increasingly apparent that distinct fibrilization conditions can produce different types or ‘strains’ of Aβ aggregates with different physicochemical properties (**Figure 2** [A])^113^. The concept of strains of protein aggregates originated from distinct conformers of disease-associated prion proteins underlying prion diseases, including Scrapie disease in sheep (the first known prion disease), hence their general designation as PrP^Sc^114^^. Different strains of PrP^Sc^ can not only be distinguished based on physicochemical properties but may also exhibit characteristic disease phenotypes, such as differences in the rate of disease progression^114^. The strain concept has since been extended to other neurodegenerative diseases, including α-SN aggregates in synucleinopathies, and Aβ and tau aggregates in AD^114^. Several techniques have been applied to characterize strains of Aβ aggregates, including (i) histological analyses based on luminescent probes that display distinct spectroscopic signatures when bound to different conformations of Aβ^115^, and (ii) biochemical assays that characterize aggregates based on their relative resistance to chaotropic agents^116^. Structural analyses of synthetic Aβ fibrils revealed that Aβ strain differences manifest as conformational differences within the fibril structure^113^. These analyses are typically based on electron microscopy or atomic force microscopy, solid-state NMR, or hydrogen/deuterium exchange^117^. More specifically, structural polymorphisms may manifest as variations in the number of protofibrils that constitute the mature fibril, orientation and arrangement of protofibrils, or conformations and arrangements of Aβ monomers within the protofibril structure^117^. In addition, Aβ aggregates may exhibit distinct biological properties, including differences in cellular toxicity when applied to neurons^113^, or, as elegant inoculation studies with bigenic Tg (APP23:Gfap-luc) mice have uncovered, may be distinguished by their incubation periods or disease pathology^116^. The existence of Aβ strains may explain some of the clinical heterogeneity observed in AD and, therefore, efforts to more fully characterize their properties and occurrence may prove beneficial for our understanding of AD.

Mass spectrometric analyses of senile plaques from AD patients revealed, in addition to aggregated Aβ, many other misfolded protein constituents in senile plaques^118^. Interestingly, these non-Aβ plaque constituents comprise several amyloidogneic peptides, including α-SN, cytostatin C, and tau^118^. Moreover, in addition to Aβ_1-42_, senile plaques also contain other Aβ products, such as Aβ_1-40_, Aβ_4-42_, pyroGluAβ_3-42_ etc^119^. Studies on Aβ aggregation have begun to account for this additional complexity and increasingly interrogate, e.g., through cross-seeding and co-aggregation experiments, how the presence of other constituents affect the spatial and temporal dynamics of Aβ aggregation.

### 6.2 Cross-seeding

Aβ aggregation follows three stereotypical phases: It begins with a nucleation phase, which can be of variable length but typically is fairly reproducible under a given set of experimental conditions, followed by a relatively short elongation phase, and an extended plateau phase^120^. Aggregation can be accelerated by seeding the elongation reaction with preformed oligomers or fibrils, which can act as nuclei for elongation (**Figure 2** [B]). This seeding can occur with homologous seeds or heterologous seeds, the latter being composed of oligomers or fibrils of a protein that can promote the aggregation of another amyloidogenic protein^120^. Because cross-seeding requires a certain thermodynamic compatibility of preformed seed templates and possible conformational conversions of the substrate, only few amyloids are capable of cross-seeding Aβ^121^. For example, bi-directional cross-seeding has been observed *in vitro *for α-SN and Aβ^122^, IAPP and Aβ^123,124^, as well as PrP and Aβ^121,125^. Sometimes cross-seeding can be unidirectional only. For instance, Aβ aggregates were observed to cross-seed Tau aggregation but Tau aggregates were unable to cross-seed Aβ^120,126^.

Evidence of cross-talk between misfolded proteins has also been observed in patients with protein misfolding diseases (PMD)^120^. Patients with PMD often have multiple misfolded proteins simultaneously existing within their bodies. For example, approximately half of all AD cases are characterized postmortem by Lewy body pathology, in addition to senile plaques^127^. Epidemiological studies have also suggested that having one PMD may be a risk factor for developing another^120^. An example is the link that has been observed between Type 2 diabetes and AD, where patients with Type 2 diabetes have an increased risk of developing AD, and AD patients have a higher incidence of islet amyloidosis compared to healthy individuals^120^. Cross-seeding provides a potential mechanistic explanation for the widespread observation of individuals presenting with multiple PMD.

### 6.3 Co-aggregation 

Based on these observations, it is not surprising that amyloidogenic proteins have been observed to interact and influence each other’s nucleation kinetics and the nature of aggregates formed in various *in vitro* paradigms. For example, when monomeric Aβ_1-42_ and the protein neuroserpin, a serine protease inhibitor, are co-incubated, Aβ aggregation occurs faster, is off-pathway, and generates non-toxic Aβ oligomers^128^. Similarly, *in vitro *co-aggregation of Aβ and α-SN monomers led to the formation of hybrid pore-like oligomers^129^. However, some amyloidogenic proteins have been shown to exert an inhibitory effect on Aβ aggregation. For example, co-incubation of Aβ_1-40_ with cystatin C, and co-incubation of Aβ_1-40_ or Aβ_1-42_ with PrP^C^ inhibited Aβ fibrillization^121^.

In AD, higher Aβ_1-42_:Aβ_1-40_ ratios seem to correlate with more aggressive presentations of the disease^54^. To better understand how Aβ_1-42_:Aβ_1-40_ ratios may influence disease, co-aggregation studies were performed with different Aβ isoform ratios^54^. Curiously, these studies demonstrated that Aβ_1-42_ accelerated Aβ_1-40_ aggregation and, conversely, Aβ_1-40_ inhibited Aβ_1-42_ aggregation^54,121,130^. Moreover, although Aβ_1-40_ and Aβ_1-42_ were shown to interact during primary nucleation steps, they eventually self-assembled into single molecular species fibrils, instead of co-assembly into hybrid, mixed isoform fibrils (**Figure 2** [C])^130^. This data is consistent with interpretations of atomic models of Aβ_1-40_ fibrils and Aβ1-42 fibrils elucidated by solid-state NMR. In these models, Aβ_1-40_ and Aβ_1-42_ fibrils are characterized by distinct and incompatible structural motifs. As discussed previously, Aβ_1-42_ adopts a distinct triple-β motif, where there is a critical salt bridge between the Ala42 residue with Lys28. Aβ_1-40_ cannot adopt this triple-β motif due to the lack of theAla42 residue at its carboxyl terminus^92^.

## 7. Functional amyloids

So far we have highlighted a body of literature that establishes the significance of amyloid forming proteins for the etiology of dementias, emphasizing the role of oligomeric forms of Aß in AD^131,132^. In subsequent sections, we will present and discuss recent evidence of an interaction between Aß and somatostatin (SST), a functional amyloid. A brief summary of where functional amyloids can be found seems warranted before introducing SST and its interaction with Aß.

### 7.1 Evidence for the existence of amyloids outside of disease

Mature amyloid aggregates play important functional roles in several cellular contexts, participating in fundamental biological processes in a wide range of organisms^10,132,133^.

The characteristic cross-b sheet quaternary structure of mature amyloids endow amyloid aggregates with unique physicochemical properties, including a yield-strength approaching that of steel^134^, and a resistance to protease-mediated degradation, and detergent-mediated solubilization^10^. Further, amyloid aggregates possess several surfaces for strong, selective binding to other molecules, possible due to the presence of both hydrophobic and hydrophilic interfaces^133^, and the high density and repetitiveness of their building blocks^133^. These properties together enable amyloids to play significant biological roles.

To showcase the breadth of biological processes^10^, which involve amyloid aggregates, we will briefly outline roles in long-term potentiation, defense against bacterial and viral infections, and in the synthesis of melanin, before discussing the significance of amyloid storage in the context of the regulated secretion of peptide hormones.

First shown in sensory neurons of the sea slug, *Aplysia*, overexpression of cytoplasmic polyadenylation element binding protein (CPEB), a regulator of translation, leads to the formation of intracellular puncta^135^. These puncta are composed of amyloid aggregates of CPEB evidenced by positive ThT staining and their responsiveness to detection by an antibody specific to the amyloid form of CPEB (Aß454). Exposure of sensory neurons to serotonin increases the rate of CPEB amyloid aggregation, thereby counteracting the short half-life intrinsic to CPEB. This elegant conformational transition allows the protein to be present long enough (i.e., minimally 72 hours) to enable LTP^136^. Thus, only through amyloid aggregation can CPEB acquire the long-term stability necessary to serve its intended purpose.

The strong, selective binding ability of amyloids is what enables amyloid aggregates of human defensing 6 (HD6) protein to play important roles in defending the body against invaders. When in the presence of pathogens, monomers and dimers of HD6 exhibit no inherent antimicrobial activity, in contrast to the five other members of the human defensin protein family^137^. Instead, HD6 monomers bind to molecular structures on the surface of pathogens and serve as seeds for the building of complex networks of amyloid fibrils known as nanonets that surround the invading pathogen^138,139^. Once encapsulated, pathogens can no longer access points of entry into the circulatory system, and are prone to attack by other components of the immune system.

Amyloids of the mitochondrial antiviral-signaling protein (MAVS) defend against viral infection using a different approach^140^. When a virus has infected a cell, the cytoplasmic retinoic acid-inducible gene 1 receptor (RIG1) binds to both the viral RNA, and to MAVS. This interaction with MAVS stimulates the assembly of MAVS monomers into large amyloid aggregates on the mitochondrial surface. MAVS amyloids then activate a variety of signaling molecules in the cytoplasm that serve as connection points for signaling events related to the cellular anti-viral response.

Melanin, a polymer expressed in the skin and retinal pigment epithelium of the eye, protects neighboring cells from invading pathogens, small molecules, and exposure to UV radiation^141^. It is hypothesized that amyloids of a fragment of premelanosome protein (Pmel17), known as Ma, serve as a scaffold for the synthesis of melanin from indole-5,6-quinone (DHQ) in melanosomes. In support of this model, melanosomes contain amyloids, evidenced by their positive staining with Congo Red and thioflavin S, as well as their resistance to solubilization with detergents.

### 7.2 Secreted peptide amyloids

In mammalian cells, some secretory proteins are released constitutively, while others are stored at high concentrations in secretory granules, and released only in response to specific stimuli^142-145^.

The ability to transiently store proteins or peptides in secretory granules at high concentrations, combined with the ability to trigger their controlled release, can provide critical advantages relative to alternative constitutive release pathways. Amyloids have unique properties that make both of these possible; packing in a format that essentially is devoid of water not only provides an exquisite space management solution but also makes them somewhat inert toward inadvertent degradation during extended storage phases. Moreover, this tight packing combined with encapsulation of hormone amyloids in secretory granules reduces the risk of potentially toxic effects of hormones by minimizing their ability to interact with other molecules^11^. Indeed, there is now compelling data in support of the conclusion that amyloid aggregates represent the predominant format for compact storage in secretory granules; secretory granules purified from AtT-20 cells stain positively when probed with anti-amyloid antibodies or the b-sheet-specific dyes ThT and Congo Red^11^. Taken together, the transient storage of hormones as amyloids is well tolerated and poses a negligible burden on a cell’s viability.

How exactly amyloid aggregates are formed and are being dissolved upon their release is still only partially understood. At least in some paradigms, other factors might facilitate these processes. A recent study has implicated protein members of the heat shock pathway in the regulation of amyloid dynamics^146^. The study proposes that in the presence of stimuli, proteins with a specific domain, referred to as the amyloid converting motif (ACM), are recruited to intracellular amyloid-rich aggregates known as A-bodies^146^. Once the stimulus dissipates, heat shock proteins mediate the disaggregation of amyloid proteins. Disaggregation analyses of amyloids composed of corticotropin releasing factor (CRF) demonstrate that, upon their secretion, amyloid aggregates of this hormone release catalytically active hormone monomers^11^. A sustained release of active monomers is also observed following the release of amyloids comprised of gonadotropin releasing hormone (GnRH)^147^. Taken together, these results corroborate the view that the amyloid structure might indeed be uniquely suited to the storage and sustained release of a subset of amyloidogenic hormones.

In summary, their unique properties enable amyloids to play significant functional roles in a wide variety of cellular contexts. Several hormones are stored and released as amyloids and it is likely that specific conditions, co-factors or additional proteins must be present for amyloid aggregation, storage, and dissociation to occur.

## 8. Biogenesis and physiological function of SST and CST

SST is a regulatory neuropeptide produced by neuroendocrine, inflammatory, and immune cells throughout the body, with high abundance in the CNS, peripheral nervous system, the pancreas, and the gut^148^. It was first identified as a GH-releasing inhibitory factor in the 14-amino acid form, SST-14^149^, followed by the subsequent discovery of SST-28 containing additional 14 amino acids at the N-terminus^150^. Both SST-14 and SST-28 are bioactive and are generated from the same inactive precursor protein, preprosomatostatin (PPSST)^151^. SST-14 is predominantly present in pancreatic islets, stomach, and peripheral neural tissues, while SST-28 is the dominant form in intestinal mucosal cells and muscles. In the brain, SST-14 accounts for about 70% of all SST-like immunoreactivity and SST-28 contributes 25% of that^148^.

### 8.1 Biogenesis of SST

SST is initially translated into the 116-amino acid PPSST by ribosomes of the rough endoplasmic reticulum (ER)^152^. Upon entering the ER, the N-terminal signal sequence of PPSST is co-translationally cleaved, resulting in the 92-amino acid prosomatostatin (PSST). Further processing of PSST occurs in the *trans*-Golgi network (TGN)^153,154^ and involves cleavage by enzymes belonging to a family of mammalian subtilisin/kexin-related, Ca^2+^-dependent serine proteinases, or precursor convertases (PCs)^151^. Whereas SST-14 is generated by dibasic cleavage at an Arg-Lys residue pair, monobasic cleavage at an upstream Arg residue produces SST-28^155^. Both SST-14 and SST-28 circularize by forming an internal disulfide bond between Cys3 and Cys14 (based on SST-14 amino acid counts). An additional N-terminal cleavage of PSST results in the decapeptide PSST (1-10), which has no known biological activity.

Early in its biogenesis SST is directed to the regulated secretory pathway (RSP) where it is stored in secretory granules until its release in response to appropriate stimuli^156-158^

SST is one of several peptide hormones (see Section 6) that forms β-sheet-rich amyloid-like aggregates in the secretory granules^11^. *In vitro* studies established that under non-denaturing conditions, at around pH 5, cyclic SST-14 can self-assemble into laterally associated nanofibrils that exhibit amyloid characteristics and are ultrastructurally composed of fixed β-hairpin backbones^159^. The rate of fibrilization of SST-14 is accelerated and the minimal concentration favoring amyloid formation is lowered in the presence of physiological salt concentrations. Removing the disulfide bond results in a linearized non-cyclic SST-14 (ncSST-14), which forms aggregates even more readily than native SST-14, possibly because it displays a higher conformational flexibility^160^. The self-assembled aggregates of ncSST-14 are stabilized by highly organized interpeptide hydrogen bonds (H-bonds), resulting in a relatively higher resistance to thermal and guanidine hydrochloride-mediated denaturation, and a slower rate of fibril reversing to monomers when exposed to physiological pH, compared to native SST-14.

It has been proposed that the propensity of hormone peptides to aggregate serves as a sorting mechanism for entering secretory granules in the RSP^11^. Amyloid aggregation of SST might be initiated spontaneously at somewhat acidic pH in the Golgi apparatus when the hormone concentration is above a critical value. Because prohormones aggregate more slowly than the mature peptide products, the rate of aggregation of this neuropeptide hormone might also be regulated by the endoproteolytic processing of the prohormones. Once the process has been initiated, nascent aggregation states might be further condensed by the high hormone concentration and the more acidic pH present in secretory granules. In the process, which is facilitated by the presence of heparin^161^, amyloid aggregates s omehow attract membrane lipids to surround the hormone aggregates, thereby forming the nascent granule, which subsequently is severed from the Golgi cisterna^162^. Once formed, secretory granules represent a stable depot for long-term storage of peptide hormones.

### 8.2 SST release

The release of SST from neurons and peripheral SST-secreting cells involves the fusion of the secretory granules with the plasma membrane and is regulated by a variety of physiological stimuli that cumulatively lead to the depolarization of the cellular membrane, followed by Ca^2+^ influx through voltage-sensitive Ca^2+^ channels. Several ion channels are involved in the membrane depolarization^163^. Addition of veratridine, a depolarizing K^+^ and Na^+^ channel agonist, has been shown to trigger SST secretion from the rat hypothalamus^164^. A role of Ca^2+ ^in the process is apparent based on the observation that the SST release in response to K^+^ channel activity is proportional to the Ca^2+^ concentration in the medium. Besides, both the removal of Ca^2+^ from the medium or the addition of blockers of voltage-sensitive Ca^2+^ channels prevented the veratridine-mediated SST secretion. Several substances that trigger the controlled release, generally referred to as secretagogues, have been identified *in vitro* and *in vivo*. Some of them act relatively broadly, while others exhibit exquisite specificity and sensitivity when added to SST-releasing tissue or cells^151,165^. In light of SST’s early recognized role as a GH inhibitory hormone, it is not surprising that GH was later observed to stimulate SST secretion in the hypothalamus through feedback regulatory loops^165^. SST release can also be modulated by other neurotransmitters, including dopamine (DA), opiate, gamma aminobutyric acid (GABA), acetylcholine (ACh), norepinephrine (NE), and substance P, which were observed to exert either stimulatory or inhibitory effects on the release of SST^151^. Intriguingly, glucocorticoids were documented to increase SST release at low doses but became inhibitory at high doses^166^. Moreover, functionally related inflammatory factors were seen to act differentially on the release of SST. Whereas interleukins 1, 6, and 10, interferon-γ, and tumour necrosis factor-α stimulated SST release, transforming growth factor-β blocked its release^148^.

### 8.3 Physiological function of SST in the brain

Because of its broad distribution and crosstalk with cytokines and neurotransmitters, SST is thought to modulate endocrine, immune and CNS functions. In the brain, SST works as a neurotransmitter that affects neuronal responses to synaptic inputs by presynaptic inhibition. Specific examples of its activity are the SST-mediated reduced secretion of DA from the midbrain or its inhibitory effect on the release of NE, thyroid-releasing hormone, and corticotrophin-releasing hormone from the hypothalamus^151^. In the dentate gyrus, SST inhibits glutamate release and exhibits long-lasting effects on glutamatergic synapses, reducing the likelihood of generating LTP^167^, thereby elevating the activation threshold required for the acquisition of new memories. The benefit of SST release in this context might manifest in an improved ability to eliminate irrelevant environmental cues^167^. Corroborating this notion, administration of SST or its analog agonists was described to improve the performance of rodents in certain cognition paradigms, such as avoidance tasks and shuttle box learning tests^168^. SST also increased locomotor activities, and conversely, treatment with SST-depleting agents, cysteamine and pantethine, impaired these cognitive and locomotor functions. SST has also been implicated in several neurological diseases, and reduced level of SST in the brain and the cerebrospinal fluid has been observed in patients with Alzheimer disease, Parkinson disease, Schizophrenia, and Huntington disease^168^. All of the above points toward a positive role of SST in memory, learning and locomoter performance. However, the situation is more complex: Although intrahippocampal cysteamine injection impaired spatial learning, it accelerated the acquisition of the bar-pressing task in mice, consistent with a role of SST in some but not other learning and memory tasks. Even more strikingly, SST knockout mice were reported to display significant impairments in motor learning tasks but no major learning and memory defects^151^.

Cortistatin (CST), a paralog of SST originally discovered in rat brain, is encoded by a gene on Chromosome 4 that has little resemblance in its nucleotide sequence to the Chromosome 6 gene locus coding for SST^169^. Unlike SST, CST is restricted to the CNS, with highest expression levels in the cortex and hippocampus. However, analogous to SST, CST emerges from consecutive cleavages of preprocortistatin (PPCST) that produce two predominant bioactive products, termed CST-17 and CST-29. And although the two genes differ in their nucleotide sequence, cyclic CST-17 shares 11 amino acids with SST-14, including all residues understood to play critical roles for docking to SST receptors (SSTRs) (see Section 9) and, therefore, mostly acts as a natural analog of SST-14 in SSTR binding assays^169^. As expected, CST-17 also exerts inhibitory neuronal activities and modulates inflammatory responses, learning and memory, as well as locomotor functions. However, the activity profile of SST and CST is not perfectly overlapping even in regions that express both peptide hormones^170^. For instance, in contrast to SST-14, CST-14, the rat homolog to CST-17, was observed to markedly decrease rodent locomotor activities. Also unlike SST-14, CST-14 was observed to modulate sleep/wake cycles and promote slow-wave sleep^169^, probably by enhancing the hyperpolarizing activity of cortical neurons and antagonizing excitatory effects of actylcholine^171^. At least in part, these sub-specializations of their activities may reflect differences in the binding profiles and range of receptors the two peptide hormones interact with.

## 9. Evidence for direct interaction of SST and CST with Aβ

**Figure 3:**
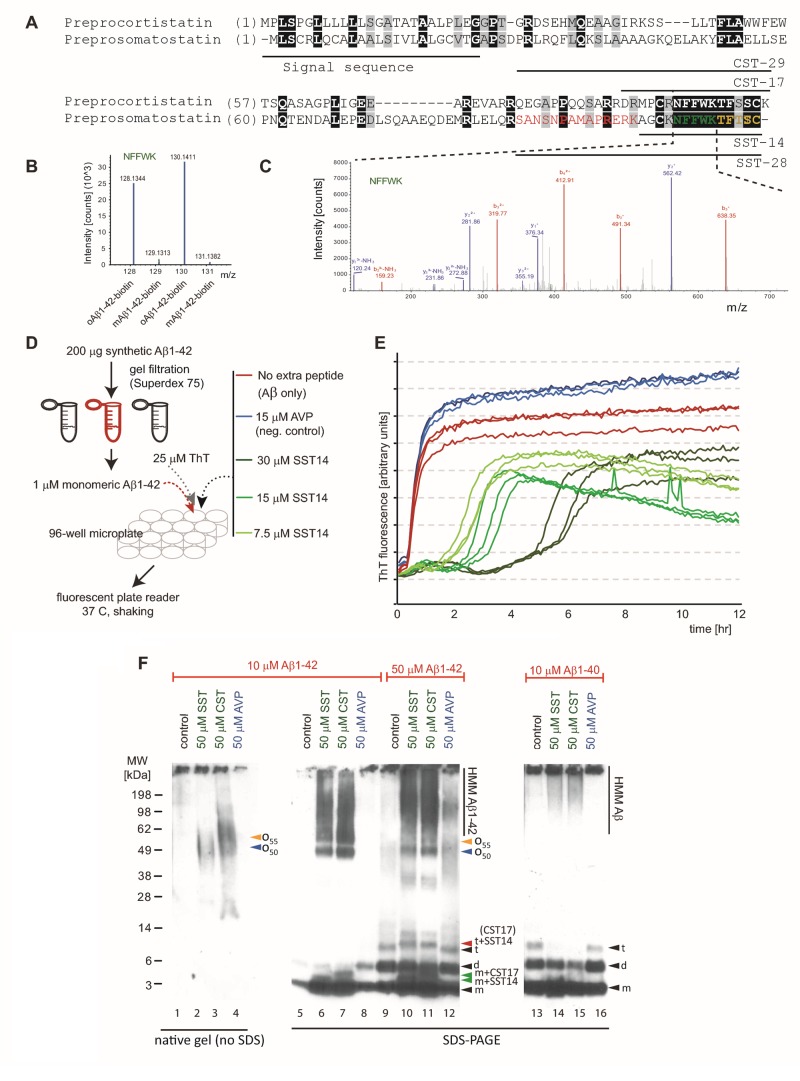
Discovery and validation of SST-Aβ interaction **A.** Sequence alignment of preprocortistatin and preprosomatostatin. The signal sequence and the boundaries of the bioactive cortistatin and somatostatin peptides are indicated by horizontal bars. Identical residues are highlighted by black background shading, and peptide sequences observed by mass spectrometry are shown in colored fonts. **B.** Expanded view of MS3 spectrum derived from ‘NFFWK’ parent spectrum (shown to the right) in interactome study based on oAβ1-42-biotin baits and mAβ1-42-biotin negative controls. In this view, the relative intensities of tandem mass tag (TMT) signature ions reflect the relative abundances of the ‘NFFWK’ peptide in side-by-side generated affinity purification eluate fractions, indicating preferential binding of SST to pre-aggregated oAβ1-42. **C.** Example tandem MS spectrum supporting the identification of the peptide with amino acid sequence ‘NFFWK’. Fragment masses attributed to B- and Y- ion series are shown in red and blue colors, respectively. **D.** Workflow of ThT-based aggregation assay. **E.** SST14 delays Aβ1-42 aggregation in ThT fluorescence assay in a SST14 concentration dependent manner. **F. **Immunoblot analyses with an antibody directed against an N-terminal Aβ epitope (6E10) reveal that CST17 (or SST14) co-assemble with Aβ1-42 into oligomers of 50-55 kDa that withstand boiling (lanes 2 and 3) but partially disintegrate in the presence of SDS. Note bands of 5-6 kDa, consistent with the existence of SDS-resistant heterodimeric complexes of mAβ1-42 and SST14 (or CST17), and the well-defined oligomeric bands of 50 and 55 kDa (lanes 6 and 7) that were observed in samples derived from the co-incubation of SST14 (or CST17) with Aβ1-42, but not Aβ1-40 (lanes 6, 7, 14, 15). Note also that signals interpreted to represent trimeric Aβ1-42, but not dimeric Aβ1-42, can be seen to migrate slower in the presence of SST14 (or CST17) but not the negative control peptide AVP (compare lanes 9 and 12 with lanes 10 and 11). Finally, intensity levels of homodimeric Aβ1-42 bands are reduced in the presence of SST14 (or CST17) (compare lanes 13 and 16 with lanes 14 and 15). Black arrowhead labeled with ‘m’, ‘d’, and ‘t’ designate bands interpreted to consist of monomeric, dimeric and trimeric Aβ1-42. Green and red arrowheads were used to label bands interpreted to represent SDS-stable heteromeric building blocks consisting of SST14 (or CST17) bound to monomeric and trimeric Aβ1-42, respectively. Elements from this image were adapted from^12^, licensed under CC BY 4.0.

To identify novel binders to oligomeric Aβ (oAβ), we recently undertook a deep interactome analysis using oAβ preparations as the bait and post-mortem human frontal lobe brain tissue obtained from individuals who died of non-dementia related illnesses as the biological source material. This unbiased approach led to the discovery and subsequent validation of SST, as a highly selective binder of oAβ (**Figure 3** [A]). More specifically, of more than one hundred proteins that co-affinity purified with the oAβ bait, the relative quantitation methodology employed, which afforded a direct comparison of candidate interactors binding to oAβ versus mAβ (**Figure 3** [B]), identified SST as the most selective oAβ binder. SST further stood out amongst other Aβ candidate interactors by its small size. For binding to oAβ to occur the latter had to be tethered to the affinity matrix through its C-terminus. Subsequent reciprocal affinity capture experiments and fluorescence resonance energy transfer (FRET) assays confirmed binding of SST to oAβ.

Utilizing ThT-based fluorescence assays, we observed that the presence of SST robustly affected Aβ-dependent ThT absorbance kinetics (**Figure 3** [D, E]), suggesting that its presence can impact Aβ amyloid formation. Finally, we documented that the presence of SST traps a considerable proportion of Aβ in an oligomeric assembly of 50-60 kDa (**Figure 3** [F]). The size of this oAβ complex is reminiscent of the aforementioned oAβ*56, a complex reported to exist in certain APP overexpressing transgenic mice^86^ and the brains of individuals afflicted with AD^88^.

## 10. Interactors of SST (and/or CST)

In an attempt to anticipate the potential significance of SST for the pathobiology of AD, it is critical to also consider SST interactions with other proteins. This section will discuss the main cellular SST receptors and their downstream signals. This information is relevant in this context because it will reveal that binding of Aβ to SST is likely to compete with SST binding to its receptors. In the next sections we will show that this scenario is not unlikely due to the spatial overlap of the respective proteins in the brain (section 10). We will return once more to SST signalling (section 11) when discussing the existence of an independent mechanism by which SST may influence Aβ levels indirectly (section 11).

### 10.1 Interactions of SST (and CST) with other proteins

SST exerts its widespread physiological effects through interaction with five SST receptors (SSTR1 to SSTR5) coded in humans by *SSTR1 to SSTR5* genes, which map to chromosomal bands 14q13, 17q24, 22q13.1, 20p11.2, and 16p13.3, respectively. SSTRs can be grouped on the basis of the structure, phylogeny and pharmacology of their expression products into two classes: SRIF1, comprising *SSTR2, SSTR3 and SSTR5*, and SRIF2, comprising *SSTR1* and *SSTR4*^172^. Out of the five human SST receptor genes only *SSTR2*contains introns and gives rise to two alternatively spliced isoforms. SSTR1 to 5 have been shown to be composed of 7 alpha-helical transmembrane domains. CST also interacts with and signals through two additional receptors which SST does not bind to: MAS-related gene X2 to increase intracellular calcium, and GH secretagogue receptor 1a to increase prolactin release^173,174^.

SST receptors are expressed in many cells throughout the body, notably in the brain, pituitary gland and the pancreas, as well as in certain tumors^148,172^. Amongst all SSTRs, the expression of SSTR2 is the highest in the CNS, with broad SST-mediated activation in hippocampal, cortical and limbic (i.e., amygdala) neuronal networks. SSTR1, 3 and 4 play more specific functional roles in the CNS but SSTR5 is expressed at low levels in the brain^175^. Therefore, SST-related deficits in the brain may be most likely mediated through loss of SSTR2 signaling^175^.

All five SST receptor are coupled to the pertussis toxin-sensitive G_i_ protein. SSTR2 and SSTR5_ ­_are additionally coupled to G_o_ and G_q_ proteins, respectively^172,176^. SST-14, SST-28 and CST-17 bind to SSTRs with nanomolar affinity, and SSTR5 is the only receptor which displays higher affinity for SST-28 over SST-14^172,177^.

**Figure 4:**
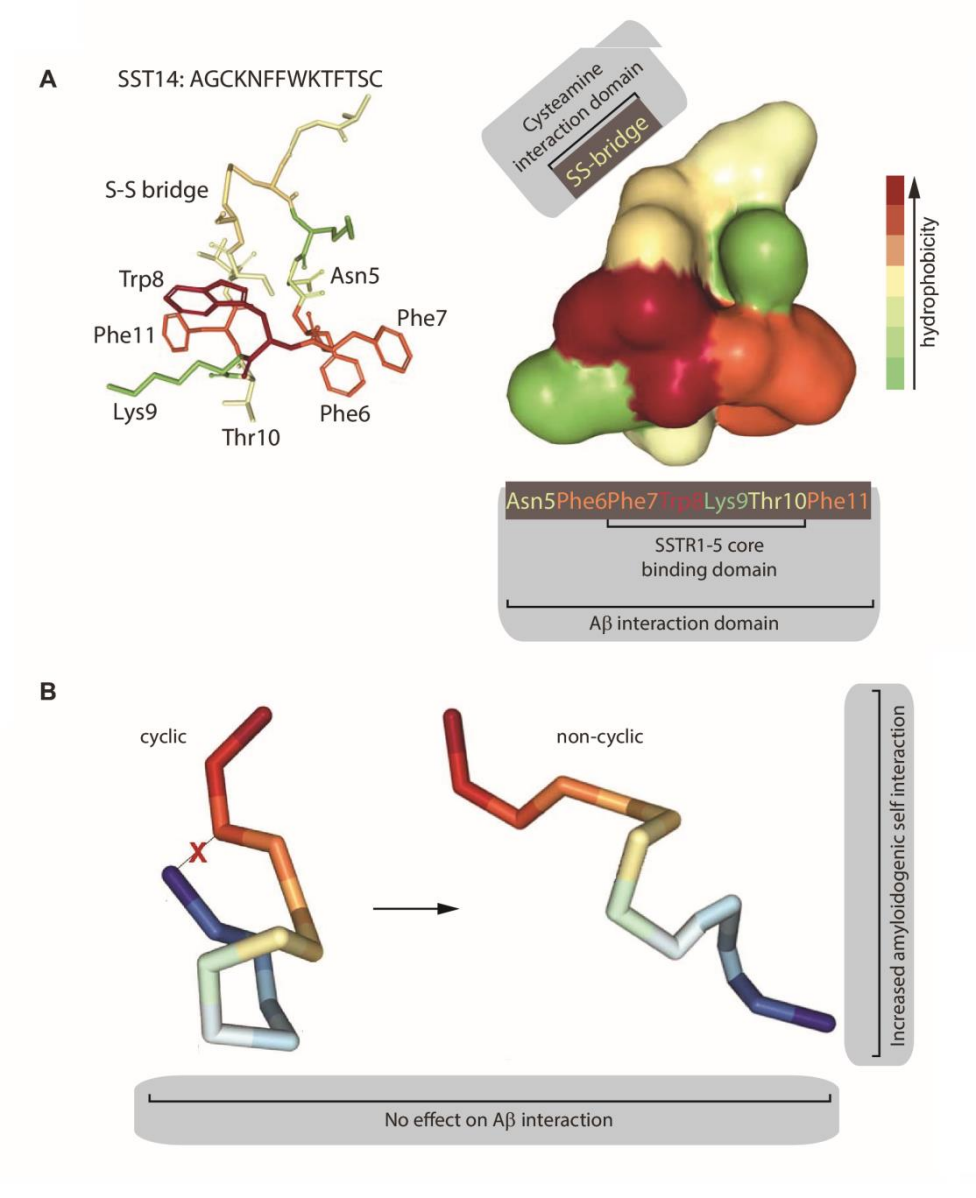
Interactions of cyclic and non-cyclic somatostatin **A.** The natural state of somatostatin is cyclic (SST14 and SST28), formed by the presence of a disulfide bridge between cysteine 3 and 14. Cyclic somatostatin is able to dock to sst1-5 receptors with high affinity through a binding epitope spanning residues 7 through 10, which represents the core region responsible for its binding to Aβ. **B.** Cyclic somatostatin can also form amyloids through self-aggregation. Cysteamine reduces the disulfide bridge, leading to a transition to non-cyclic somatostatin, which has a higher propensity to self-aggregate. The amyloidogenic binding domain in non-cyclic somatostatin is between residues 3 through 14. Adapted from the somatostatin structure in the PDB database^160^.

The internal disulfide bridge present in SST restricts the conformational freedom of SST-14 and SST-28 and contributes to ligand-receptor binding^148,160^ through its core binding epitope comprised of the sequence motif Phe-Trp-Lys-Thr (i.e., amino acids 7 through 10 in SST-14) (**Figure 4** [A])^178^. Note that a slightly broader binding epitope that spans SST

residues 5 through 11 was observed to be residues 5 through 11 was observed to be responsible for binding of Aβ to SST^12^, suggesting that interactions of SST with its receptors or Aβ are mutually exclusive. Much of what is known about SSTR binding to its receptors is the result of studies investigating SSTR2 and SSTR5. According to these studies, it is thought that the acceptor docking site on SST receptors is molded from hydrophobic and charged residues in the second extracellular loop and transmembrane domains III-VII. This interface model came into focus on the basis of data generated with multiple indirect techniques, including the use of SST analogues (e.g., octreotide), SST receptor chimeras, and site-directed mutagenesis. Through studying the binding of octreotide with SSTR2, it was discovered that Asn276 and Phe294 in transmembrane domains VI and VII stabilize the interaction with Phe7, Trp8 and Thr10 within the SST binding epitope, as well as the Cys3-Cys14 disulfide bridge. Binding is further stabilized by an electrostatic interaction between Lys9 on SST-14 and Asp137 on transmembrane domain III of the receptor^148,179^.

### 10.2 Signaling downstream of SST (and CST)

Binding of SST to the SSTR2 receptor may recruit kinases to rapidly phosphorylate its C-terminal domain at three serine (Ser341, Ser343 and Ser348) and four threonine (Thr353, Thr354, Thr356 and Thr359) residues leading to complexing with β-arrestin1^180^. It has been proposed that the G protein-coupled receptor kinase 2 and 3 (GRK2/3) is largely responsible for SSTR2 phosphorylation at these phospho-acceptor site clusters but the phosphorylation at Ser343 may also depend on protein kinase C^180,181^. Additionally, three tyrosine residues (Tyr71, Tyr228 and Tyr312) can be phosphorylated and regulate SSTR2 activation and signaling through alternative pathways, which are discussed in more detail elsewhere^182^. For SSTR5, SST ligand-activated phosphorylation occurs at a single threonine residue (Thr333) by GRK2/3 within its carboxyl tail domain^182,183^.

The phosphorylation of SSTR2 or 5 at sites exposed within the cytoplasm leads to their complexing with β-arrestin1, which in turn targets protein phosphatase 1β (PP1β) or 1γ (PP1γ) to the receptors, causing the dephosphorylation of SSTR2^184,185^ or SSTR5^185,186^, respectively. Since activation of SSTR5 is reliant on a single phosphorylation, the internalized β-arrestin-SSTR5 complex is unstable and resensitization occurs quite fast (< 10 mins), when compared to SSTR2 (approximately 30 mins). Elegant C-terminal domain swapping experiments, based on chimeric SSTR2 and SSTR5, corroborated the conclusion that the differences in their dephosphorylation rates depend upon the composition of the respective C-terminal tails^187^.

Several second messenger systems and downstream signaling pathways can be activated by SST and CST^172,176^. Here, we will restrict descriptions to SST receptor-mediated inhibition of GH secretion. In this paradigm, binding of SST to SSTR2 and SSTR5 triggers a highly integrated cellular response that cumulatively restricts cellular exocytosis, and therefore, causes decreased secretion of GH. To accomplish this outcome, SST binding directly affects three critical activities: (i) the internalized SST receptor complex inhibits adenylyl cyclase, leading to a decrease in cAMP levels. cAMP signaling normally potentiates hormone secretion through many pathways (reviewed in^188^), (ii) SST signaling potentiates K^+^ efflux channels leading to hyperpolarization of the cell and, consequently, a decreased influx of Ca^2+ ^through voltage-gated channels, and (iii) the SST receptor complex exerts a direct effect on L-type calcium channels, causing further decreases in cytosolic Ca^2+ ^influx^172^.

### 10.3 SST interactions with other small molecules and peptides

Despite continuous interest in SST and CST and extensive investigations of their molecular interactions, interactions of these cyclic peptide hormones with other small molecules or peptides have not been reported. It has been observed though that cysteamine, a precursor in the formation of coenzyme A, can effectively deplete levels of SST-14 and SST-28^189-191^. Administration of pantethine, a cysteamine precursor, also depletes SST, likely through increased cysteamine levels^192,193^. In fact, cysteamine can interact directly with SST through the breakage of the Cys3-Cys14 disulfide bridge, thereby affecting its conversion from cyclic SST (cSST) to non-cyclic SST (ncSST)^162^, and altering the kinetics of the formation and disassembly of the respective SST amyloid aggregates (**Figure 4** [A, B]) (see also Section 7). It may be expected that cysteamine can also break the internal disulfide bridge in CST but, to date, no direct interaction with cysteamine has been reported for this lesser studied paralog^194^.

Although a direct interaction of SST and amylin has not been reported, SST exposure of cells has been shown to interfere with amylin secretion, possibly through a similar mechanism as outlined above for its influence of GH release. Amylin, or IAPP, is a peptide composed of 37 amino acids which is co-released with insulin from β-cells of the pancreas to suppress blood glucose levels. Amylin is thought to play a role in the pathogenesis of diabetes, especially Type II diabetes, on the basis of data, which established that the levels for this protein are generally decreased in the disease, yet islet cells were observed to be loaded with amylin aggregates. Amylin has also been investigated in the context of AD pathogenesis because Type 2 diabetes can be a well-known co-morbidity of AD and amylin aggregates have amyloid characteristics^195^. Amylin also binds to Aβ oligomers and plaques. When its association with Aβ oligomers was studied using molecular dynamics simulations, amylin was predicted to differentially affect aggregation depending on the conformational Aβ assembly analyzed^196,197^. Finally, amylin treatment in AD mouse models decreased neuroinflammation, behavioral deficits and Aβ pathology^198,199^. Cumulatively, this body of data are consistent with the existence of an intricate intersection of the biology of SST/CST, Aβ and amylin in the pathogenesis of AD^200,201^.

## 11. Distribution and levels of Aβ versus SST (or CST) in the healthy brain

To provide insight into whether SST and CST can physically interact with Aβ peptides *in vivo*, we consider their subcellular distribution and relative localization in brain tissue, as well as their expression levels in healthy and aged brain.

### 11.1 Distribution of Abeta producing cells relative to SST or CST

Because many studies failed to explicitly differentiate between Aβ and APP, and a larger number of reports are available that inform about the localization of APP, spatial information for Aβ will reflect both reports on Aβ and its APP precursor. In addition, we will explore in this section the levels of these proteins in biofluids.

At the sub-cellular level, interactions between APP/Aβ, SST, CST and SSTRs could most readily occur in the ER, Golgi and at the plasma membrane, since all of these proteins traverse the secretory pathway^202-205^. Additional interactions are most plausible following endocytic uptake of these proteins/peptides within early and late endosomes.

**Figure 5:**
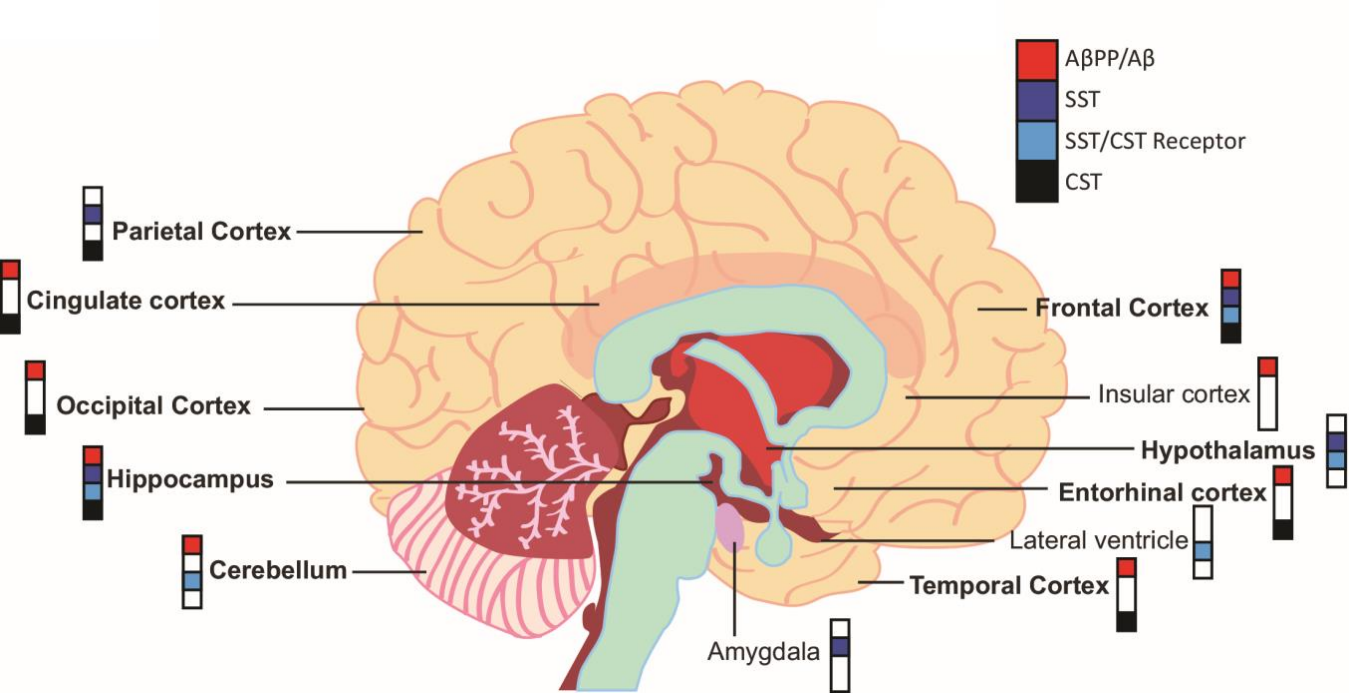
Widespread distribution of AβPP/Aβ, SST and CST across the human brain Schematic summarizing key brain areas reported to express AβPP/Aβ, SST and CST or sst1-5 receptors. Bolded labels indicate areas of the brain known to express at least two of the proteins/peptides of interest.

On the gross anatomical level, Aβ is expressed in several regions across the cerebral cortex, and may, on the basis of immunohistochemical data, predominantly localize to the temporal, insular, frontal, occipital, entorhinal and cingulate cortices^206^. A more recent immunofluorescence analysis corroborated the earlier reported relative distribution of APP within the cerebral cortex, and additionally mapped this protein to the cerebellum and hippocampus (**Figure 5**)^202^. The distribution of SST in the brain broadly overlaps with areas in which APP/Aβ is found, including the cerebral cortex (parietal and frontal cortices) and the hippocampus but may also extend to the hypothalamus and amygdala^202,207,208^. In addition to regions expressing SST, SST receptors have also been found in the cerebellum and lateral ventricles^202^. CST has been detected in the cerebral cortex (frontal, parietal, temporal, occipital, cingulate and entorhinal cortices) and hippocampus^194^. Additionally, a CST-specific receptor (MRGPRX2) has been localized to the cerebellum^202^. In regions with overlapping CST and SST expression (cerebral cortex and hippocampus), CST was observed in ~25% of cells that also expressed SST, indicating that these two peptides are often produced in the same cell^194^. Although it has yet to be directly demonstrated that APP/Aβ can be produced in the same cell as SST/CST, this scenario is also plausible given the overlapping distribution of these peptides in the cerebral cortex and hippocampus. The tentative conclusion from these incomplete mapping analyses is that there seems to be substantial overlap in the localization of APP/Aβ, the SST or CST cyclic peptides and their receptors, including in AD pathology-relevant regions, such as the hippocampus.

### 11.2 Brain and CSF levels of Aβ and SST 

Although the levels of Aβ throughout brain tissue has been measured at low picogram Aβ per mg total brain protein, dependent on brain region^209^, such human brain data remain elusive for SST, and even less is known for CST in this regard. In the CSF of healthy individuals (mean age ~60-70), Aβ_40_ has been quantified at levels ranging from 6.38 ng/mL to 10.7 ng/mL^210,211^. Levels of Aβ_42_ were independently reported in three studies to be considerably lower (i.e., 5 to 50 times) at 135 pg/mL, 325.5 pg/mL and 1.37 ng/mL^210-212^. Levels of SST in the CSF appear to be even lower than both Aβ_40_ or Aβ_42_ at concentrations of 24.8 pg/mL to 62.4 pg/mL in healthy individuals^213,214^.

The levels of Aβ and SST change during the normal aging process. Aβ levels increase in the brain and decrease in the CSF^215^. It has been proposed that this observation may reflect defective clearance from the brain, thereby increasing the chance for Aβ plaque formation in the brain^216^. The levels of SST mRNA decrease with age in the frontal, parietal and temporal cortices and hippocampus, as evidenced by studies with humans and several model species, including rats and non-human primates^215-219^. Given that the expression of Aβ, SST and CST is overlapping in several regions of the brain, including the cerebral cortex and hippocampus, it is conceivable that these peptides encounter each other *in vivo*.

## 12. Changes to the expression or down-stream signaling of SST/CST in AD

### 12.1 Levels of SST in AD, during aging and in neurological disorders

A reduction in SST-like immunoreactivity was amongst one of the earliest biochemical differences documented in the brains of individuals who succumbed to AD^220^. Since then, several studies, including work published as recently as in 2009^221^, have confirmed that the aforementioned age-dependent decrease in SST levels is exacerbated in AD patient brains (reviewed in^175^). Although documentation of age-dependent changes in CST expression is scarcer, CST mRNA expression levels were reported to increase with age, specifically within the hippocampus and dentate gyrus^222^. Interestingly, this increase was attenuated in transgenic mice that overexpress APP and are known to exhibit Aβ deposition.

CSF Aβ_40_ levels remain relatively unchanged in AD patients compared to age-matched healthy controls, however, CSF Aβ_42_ levels were shown to decline by 36% to 82%^210-212,223^. Similarly, SST levels in the CSF were repeatedly observed to decline by 41% to 76% in AD patients when compared to age-matched healthy controls^213,214^. However, changes to SST levels are not uniquely associated with AD, as other brain pathological conditions, ranging from epilepsy to neuropsychiatric disorders are associated with changes in the levels of SST or its receptors, or alterations to the density of SST-expressing neurons^224^. More specifically, loss of SST is a well-documented observation in models of epilepsy and traumatic brain injury^225^. In schizophrenia, decreased levels of SST were reported in the CSF, reduced *SST* gene expression was documented in the prefrontal cortex, and lower numbers of SST-positive neurons were observed in the entorhinal cortex and hippocampus^226^. In contrast, in bipolar disorder, CSF SST levels were reported increased, while SST-expressing interneurons were decreased in the hippocampus and entorhinal cortex^227^.

### 12.2 Evidence for genetic linkage of the SST gene to AD

At the genetic level, the human *SST* gene at its chromosomal band 3q27.3 emerged in a GWAS, undertaken with a cohort of samples collected in Finland, as a genomic region (defined by SNP rs4988514) that might modulate the risk to acquire late-onset AD. Because the effect was most pronounced in a subcohort of samples characterized by the APOE ε4-allele, the authors suggested that SST may interact with the APOE ε4-allele to increase the risk of AD^16^. Interestingly, the same SNP locus was also linked to increased AD risk in a Chinese GWAS. However, due to its mapping to the 3’ un-translational region of the *SST* gene, the authors favored the mechanistic interpretation that this linkage may indicate altered binding of factors that regulate translation, thereby ultimately affecting gene expression^17^.

### 12.3 Signaling downstream of SST influences Aβ degradation

Elegant experiments undertaken with primary neurons prepared from *Sst*^-/- ^mice revealed a dose-dependent effect of SST exposure on neprilysin activity^15^. Neprilysin, aka membrane metallo-endopeptidase (MME), is synthesized in the soma of neurons, from where it is passaged through the secretory pathway and axonal transport vesicles to presynaptic terminals. The active site of neprilysin faces the luminal/extracellular side of membranes^221^. Several lines of investigation point toward a role for neprilysin in the pathobiology of AD: In a seminal early study neprilysin emerged from an inhibitor screen as an endoproteinase that prevents the degradation of Aβ^228^. More specifically, injection of radiolabeled, and therefore traceable, Aβ_1-42_, into rat hippocampi led to its rapid degradation (half-life of 15 to 20 min) that could be blocked by the infusion of the neprilysin inhibitor thiorphan, resulting in the appearance of amyloid deposits in 30 days. Aβ_1-42_ is cleaved by neprilysin at its C-terminus between glycine residues 37 and 38, creating a longer N-terminal fragment and a short diagnostic Aβ_38-42_ peptide. Further characterization revealed that for neprilysin to be able to effectively degrade Aβ it need to be post-translationally glycosylated and have reached the cell surface^15^. The original report was corroborated by several other studies and the neprilysin paralog, neprilysin 2, aka membrane metalloendopeptidase like 1 (MMEL1), has since also been shown to degrade Aβ^229^.

To date, possible effects of CST on neprilysin levels have not been studied, however, it has been observed that expression of CST in the cerebral cortex and hippocampus by GABAergic neurons induces tau phosphorylation at Ser262^230^, a phosphor-acceptor site that has been shown to regulate tau binding to microtubules and is phosphorylated in AD^231,232^.

## 13. Evidence for co-localization of SST and CST with plaques and NFTs

As early as in 1985, a co-deposition of SST and Aβ amyloid was reported^1^. Using post-mortem AD brains, this study determined that 20% of neuritic Aβ plaques in the cortex and hippocampus, and up to 50% of plaques in the amygdala contained SST-positive immunoreactivity^233^. The corresponding photomicrographs were analyzed for overlap in signals derived from thioflavin-S, a stain recognizing the common β-sheet structure present in Aβ and other protein aggregates^234^, and SST immunoreactivity conjugated to peroxidase (**Table 1**). The authors suggested that the reported percentages may be an underestimation, since serial sections were not used for quantification, and the penetration of the SST-targeting antiserum through a 50 µm section may have been suboptimal^233^. The antiserum used was also responsive to SST precursors, such as preproSST and proSST, which presents the possibility of cross-reactivity with these precursors of mature SST neuropeptide. A follow up paper by the same group found somewhat reduced levels of 5% colocalization between SST and/or substance P with neuritic Aβ plaques in the neocortex, hippocampus, and amygdala^235^. The authors attributed the differences in overlap, relative to their initial report, to the existence of regional concentrations of SST but also concluded that the alkaline phosphatase immunoprecipitation protocol used in this later study is less sensitive than the peroxidase protocol used in the first study^235^.

**Table 1:** Evidence for colocalization of SST with amyloid or tau

Model	Staining Method	Additional Notes	Reference	Colocalization Found
Colocalization of Somatostatin and Aβ Plaque				
Human brain (cortical regions)	IHC	20-50% of neuritic plaques contain SST-positive profiles	(Armstrong et al., 1985)	Yes
Human brain (hippocampus, amygdala, neocortex)	IHC	Only 5% of 12,000 plaques were found to contain SST or substance P	(Armstrong et al., 1989)	Yes
Human brain (anterior olfactory nucleus)	IHC & IF	65.43% of SST staining colocalized with Aβ, 19.75% co-localized with Aβ and tau	(Saiz-Sanchez et al., 2010)	Yes
Human brain (piriform cortex)	IHC & IF	43% SST cells colocalized with Aβ, 24% colocalized with Aβ and tau, 25% not colocalized with pathology	(Saiz-Sanchez, De la Rosa-Prieto, Ubeda-Banon, & Martinez-Marcos, 2015)	Yes
Colocalization of Somatostatin and Tau				
Human brain (hypothalamus)	IHC & IF	Not quantified, but mainly seen in older cohort. Significant inverse association between tau and SST staining χ2 p<0.001	(van de Nas, Konermann, Nafe, & Swaab, 2006)	Yes
Human brain (anterior olfactory nucleus)	IHC & IF	2.5% of SST staining colocalized with tau	(Saiz-Sanchez et al., 2010)	Yes
Aged JNPL3 (hippocampus)	IHC & IF	Colocalization not exhaustive in CA1 and DG region evaluated	(Levenga et al., 2013)	Yes
Human brain (piriform cortex)	IHC & IF	7% of SST cells colocalized with tau	(Saiz-Sanchez et al., 2015)	Yes
THY0-Tau22 mouse (olfactory and cortex) & human brain (anterior olfactory nucleus)	IHC & IF	No colocalization seen at 4, 8, 15 months of age, or in human brain	(Martel et al., 2015)	No

During the subsequent two decades, interest in the field shifted to gene products that emerged from genetic analysis, and few studies explored the colocalization of SST and Aβ^236^. One notable study from that time period reported that the Alz-50 antibody, which was routinely used to detect conformational epitopes within AD tau^237^, also reacts with SST-containing neurons in the hypothalamus of non-demented control subjects^238^. Regrettably, however, the authors were not able to reveal the nature of the molecule stained.

In 2010, a study revisited the colocalization of Aβ and SST in AD post-mortem brains, using anterior olfactory nuclei as specimens^239^. Here, 65% of SST-positive cells were associated with Aβ plaques, and an additional 20% were associated with both Aβ and tau aggregates. The study made use of several technical advancements (relative to the 1985 report), i.e., it was based on confocal microscopy and was based on an Aβ-specific antibody (as a more precise alternative to thioflavin-S) and a purified SST-directed antibody (as opposed to an SST-reactive antiserum). In 2015, the same group repeated this analysis in the piriform cortex of post-mortem AD brains^240^. In this assessment, 30 sections were sampled from 10 AD brains, making this one of the most ambitious analyses in this area to date. The study reported a 43% colocalization of SST-positive cells with Aβ plaques, and a 24% colocalization of SST-positive cells with both Aβ and tau, thereby overall concluding that SST colocalization with Aβ is more pronounced in the anterior olfactory nucleus than the piriform cortex.

The literature also offers some evidence of colocalization between SST-positive cells and tau aggregates, although multiple sources have reported that the association of SST with tau is less frequent than its co-localization with Aβ plaques. One of the first papers to describe this limited colocalization analyzed the nucleus tuberalis lateralis of the hypothalamus^241^. For this study, 28 AD post mortem brains were used, which covered various Braak stages of pathology. The tau-specific AT8 antibody was used to identify hyperphosphorylated tau aggregates, and SST was also identified using an SST-specific antibody. Colocalization between SST and tau was demonstrated visually but not quantified. The authors stated that colocalization was found almost exclusively in older AD patients (>70 years of age) of Braak stages V-VI, as opposed to younger AD patients (40-59). There also was a significant inverse correlation noted between tau staining density and SST staining density.

In the aforementioned 2010 study that investigated Aβ and SST colocalization in the anterior olfactory nucleus, SST and tau colocalization was quantified^239^. Relative to the co-localization with Aβ, the percentage of SST cells that colocalized with tau (2.5%) was approximately 25-fold less. Because the percentage of SST-expressing cells neither colocalized with Aβ nor tau (12.4%) exceeded those that exhibited tau colocalization, the authors concluded that SST expression is not significantly associated with tau pathology.

Moving to rodent models, SST and tau colocalization was quantified using the aged JNPL3 mouse model, which expresses a human mutant P301L tau gene^242^, leading to severe hippocampus-dependent memory deficits along with electrophysiological abnormalities. Serial brain sections were incubated with an SST-specific antibody and tau was identified using both the α-MC1 and α-PHF1 antibodies. MC1 is categorized as an early tau pathology marker, which is highly non-specific, while PHF1 indicates late-stage tau pathology and is considered to be tau-specific^243,244^. Colocalization between SST and tau was noted but not quantified in GABAergic interneurons of the hippocampus, specifically in the hilus of the dentate gyrus. Conversely, a study using the THY0-Tau22 mouse model of tau hyperphosphorylation reported no incidence of SST and tau localization at 4, 8, and 15 months of age^244^. In the latter study, the AT8 antibody was used to detect hyperphosphorylated tau, and tissue sections were prepared from the olfactory tubercule, piriform cortex, and entorhinal cortex. This publication also reported an absence of SST and tau colocalization in the AD post mortem anterior olfactory nucleus; however, sample sections were obtained in this case from a single AD patient^9^. The authors suggested that the discrepancy in these results when compared to other publications stems from the Thy1.2 promoter, which is used to control transgene expression in the THY-Tau22 mouse model^244^. This promoter results in mutated tau expression in pyramidal cells of the olfactory centres, with the exception of the olfactory bulb, and spares the interneuron population, a plausible explanation why SST and tau colocalization could not be detected. Most recently, the aforementioned 2015 study by Saiz-Sanchez *et al.* also investigated SST and tau colocalization in the piriform cortex^240^. Relative to the data by the same group investigating the anterior olfactory nucleus, which demonstrated only 2.5% of SST cells colocalized with tau^239^, 7% co-localization was reported in the piriform cortex^240^. This signal overlap is substantially lower than the reported percentage of SST colocalization with Aβ amyloid (43%) in the same tissue. The authors suggested that this difference may reflect a higher density of SST interneurons in the Layer III of the cortex, where tau pathology is very sparse.

Currently there is no reports that investigated CST colocalization with either Aβ or tau. However, a careful review of binding specificities of SST antisera that were used for the early SST and Aβ colocalization studies might be indicated, as there could have been some inadvertent cross-reactivity toward CST, based on the striking sequence similarity and promiscuous ligand binding of SST receptors^170^. Overall, the occurrence of SST and Aβ colocalization is profoundly more evident than a possible colocalization between SST and tau.

## 14. Conclusion

Whenever a new wave of research discoveries instills a sense that we have turned a corner in our ongoing efforts to describe a biological system or phenomenon, before long, previously unrecognized intricacies come to the fore. Here we considered the hitherto unexplored possibility that the amyloidogenic peptide SST may influence the pathobiology of AD on account of its direct interaction with Aβ. Although merely a hypothesis at this time, the robustness of the *in vitro* interaction data under near physiological conditions, paired with the spatial proximity of synaptic release sites of Aβ and SST, strengthen this model. If validated, its significance may extend beyond AD, and similar interactions and crosstalk between functional and disease-associated amyloidogenic proteins may need to be considered also in other contexts. Given the humbling and seemingly boundless complexity of biological systems, it would perhaps be surprising if close scrutiny of other paradigms would not eventually reveal this phenomenon to be widespread.

## Bullet Points

**◊** ****In addition to its well-recognized pathobiological significance, amyloid deposition serves critical roles in the temporary storage and natural function of several proteins****

**◊ Considerable overlap in the distribution of SST and Aβ in the brain has been reported**

**◊ The natural decline of SST levels in aging is more pronounced in Alzheimer disease**

**◊ GWAS analyses point toward SST as a risk locus for Alzheimer disease**

**◊ Aβ plaques form preferentially in proximity of SST-releasing neurons**

**◊** **Aβ plaques may form preferentially in proximity to SST-releasing neurons**

**◊ Functional and pathologic amyloids may not only co-exist but affect each other**

**◊ This phenomenon may extend to other pairings of functional and pathologic amyloids**
